# Phase 1 Trial of the *Plasmodium falciparum* Blood Stage Vaccine MSP1_42_-C1/Alhydrogel with and without CPG 7909 in Malaria Naïve Adults

**DOI:** 10.1371/journal.pone.0008787

**Published:** 2010-01-22

**Authors:** Ruth D. Ellis, Laura B. Martin, Donna Shaffer, Carole A. Long, Kazutoyo Miura, Michael P. Fay, David L. Narum, Daming Zhu, Gregory E. D. Mullen, Siddhartha Mahanty, Louis H. Miller, Anna P. Durbin

**Affiliations:** 1 Malaria Vaccine Development Branch, National Institute of Allergy and Infectious Diseases/National Institutes of Health, Rockville, Maryland, United States of America; 2 Johns Hopkins Bloomberg School of Public Health, Baltimore, Maryland, United States of America; 3 Laboratory of Malaria and Vector Research, National Institute of Allergy and Infectious Diseases/National Institutes of Health, Rockville, Maryland, United States of America; 4 Biostatistics Research Branch, National Institute of Allergy and Infectious Diseases/National Institutes of Health, Rockville, Maryland, United States of America; World Health Organisation, Switzerland

## Abstract

**Background:**

Merozoite surface protein 1_42_ (MSP1_42_) is a leading blood stage malaria vaccine candidate. In order to induce immune responses that cover the major antigenic polymorphisms, FVO and 3D7 recombinant proteins of MSP1_42_ were mixed (MSP1_42_-C1). To improve the level of antibody response, MSP1_42_-C1 was formulated with Alhydrogel plus the novel adjuvant CPG 7909.

**Methods:**

A Phase 1 clinical trial was conducted in healthy malaria-naïve adults at the Center for Immunization Research in Washington, D.C., to evaluate the safety and immunogenicity of MSP1_42_-C1/Alhydrogel +/− CPG 7909. Sixty volunteers were enrolled in dose escalating cohorts and randomized to receive three vaccinations of either 40 or 160 µg protein adsorbed to Alhydrogel +/− 560 µg CPG 7909 at 0, 1 and 2 months.

**Results:**

Vaccinations were well tolerated, with only one related adverse event graded as severe (Grade 3 injection site erythema) and all other vaccine related adverse events graded as either mild or moderate. Local adverse events were more frequent and severe in the groups receiving CPG. The addition of CPG enhanced anti-MSP1_42_ antibody responses following vaccination by up to 49-fold two weeks after second immunization and 8-fold two weeks after the third immunization when compared to MSP1_42_-C1/Alhydrogel alone (p<0.0001). After the third immunization, functionality of the antibody was tested by an in vitro growth inhibition assay. Inhibition was a function of antibody titer, with an average of 3% (range −2 to 10%) in the non CPG groups versus 14% (3 to 32%) in the CPG groups.

**Conclusion/Significance:**

The favorable safety profile and high antibody responses induced with MSP1_42_-C1/Alhydrogel + CPG 7909 are encouraging. MSP1_42_-C1/Alhydrogel is being combined with other blood stage antigens and will be taken forward in a formulation adjuvanted with CPG 7909.

**Trial Registration:**

ClinicalTrials.gov Identifier: NCT00320658

## Introduction

The *P. falciparum* parasite is responsible for at least 300 million acute cases of malaria each year, with an estimated 1 million deaths, most in children in Africa [1]. Morbidity and mortality caused by malaria also have significant direct and indirect effects on the economic development of malaria-endemic countries [2]. Growing drug resistance of the parasite, widespread resistance of mosquitoes to insecticide, global climate change, and increased human travel make the sustainability of the recent advances against malaria uncertain, and a vaccine that reduced mortality and morbidity secondary to *P. falciparum* would be a valuable resource in the fight against this disease. One vaccine, RTS,S, is currently entering Phase 3 clinical trials; however this vaccine has only shown 30–65% efficacy in previous field studies [3], and a vaccine with higher levels of protection is still sought.

Over time, people living in malaria-endemic areas develop immunity to clinical disease caused by *P. falciparum*, and IgG from immune adults has been shown to reduce parasite density and clinical symptoms when administered to children with clinical malaria [4], [5]. Thus, proteins expressed during the blood stage of the parasite life cycle have been proposed as good candidates for inclusion in a vaccine. Rather than preventing infections, the purpose of a blood-stage vaccine is to reduce or prevent severe illness and complications of the disease such as cerebral malaria, anemia, and renal failure. Two leading vaccine candidates are apical membrane antigen 1 (AMA1) and merozoite surface protein 1 (MSP1), both of which are expressed on the merozoite surface and are thought to be involved in invasion of red blood cells. MSP1_42_ is a 42 kDa fragment of MSP1 which undergoes secondary processing and cleavage into MSP1_33_ and MSP1_19_ [6]. Vaccination with MSP1_42_ formulated in water-in-oil adjuvants induced protection in *Aotus nancymai* monkeys experimentally infected with *P. falciparum*, and protection from high parasitemia was correlated with antibody levels and in vitro growth inhibition [7]. Another study in *Aotus* found protection to be correlated with antibody responses, and to be adjuvant dependent, with higher levels of protection seen in the groups receiving antigen with complete/incomplete Freund's Adjuvant or ISA-720 compared to that receiving antigen with AS02A [8]. A Phase 2b study of the 3D7 allelic form of MSP1_42_ adjuvanted with AS02 in children in Kenya showed that the vaccine was not protective despite high antibody levels [9].

A previous study found the FVO and 3D7 allelic proteins of MSP1_42_ to be safe but not sufficiently immunogenic when adjuvanted with Alhydrogel [10]. The novel adjuvant CPG 7909 has been found to induce high antibody responses and in vitro growth inhibition of up to 96% when added to the candidate blood stage vaccine AMA1-C1/Alhydrogel [11], [12]. In the study presented here a vaccine containing the FVO and 3D7 proteins of MSP1_42_, MSP1_42_-Combination 1 (C1), adjuvanted on Alhydrogel with and without CPG 7909, was evaluated in malaria naïve adults.

## Methods

The protocol for this trial and supporting CONSORT checklist are available as supporting information; see Checklist S1 and Protocol S1.

### Study Design

This randomized, double-blind, Phase I clinical trial was designed to evaluate the safety, reactogenicity and immunogenicity of the MSP1_42_-C1 malaria vaccine formulated on Alhydrogel®, with or without CPG 7909, in malaria-naïve adults. This study was performed under an investigational new-drug application (BB-IND-12418) reviewed by the U.S. Food and Drug Administration (FDA). The protocol was approved by the Western Institutional Review Board (Johns Hopkins Bloomberg School of Public Health) and the National Institute of Allergy and Infectious Diseases Institutional Review Board. The study was conducted at the Center for Immunization Research, Johns Hopkins Bloomberg School of Public Health. (ClinicalTrials.gov Identifier: NCT00320658)

Thirty volunteers each were enrolled into low and high dose cohorts, 40 µg MSP1_42_/Alhydrogel® and 160 µg MSP1_42_/Alhydrogel®, for a total of 60 volunteers. Within each cohort volunteers were randomized 1∶1 to receive the vaccine with or without 560 µg CPG 7909. Enrollment was staggered for safety purposes such that 10 volunteers from each cohort (5 receiving the vaccine without CPG 7909 and 5 receiving the vaccine with CPG 7909) were enrolled, vaccinated, and followed for one week before vaccination of the remaining volunteers in the cohort. Volunteers in the high dose cohort were not vaccinated until two weeks after volunteers in the low dose cohort had received their third vaccination. Volunteers were vaccinated on study days 0, 28, and 56 with a 0.5 mL intramuscular injection given in alternate arms. A window of plus or minus one week was allowed for vaccines 2 and 3. Throughout the paper, study day refers to the nominal study day.

### Participants

Healthy male and non-pregnant female volunteers, ages 18 – 50, were recruited from the metropolitan Washington DC area. Written informed consent was obtained from volunteers in accordance with the Code of Federal Regulations Title 21, Part 50. Comprehension was confirmed using a questionnaire prior to enrollment. Volunteers were excluded if they had any of the following: evidence of clinically significant disease by examination, medical history, or clinical laboratory studies; were pregnant or breast feeding; had serological evidence of HIV, chronic hepatitis B, or hepatitis C infection; history of surgical splenectomy; receipt of blood products in the last 6 months; current medication with corticosteroids or immunosuppressive drugs; history of severe allergic reaction or anaphylaxis; participation in another investigational vaccine or drug trial within 30 days of enrollment; immunization with a live vaccine in the previous 4 weeks; prior malaria infection, previous receipt of a malaria vaccine, travel to a malaria-endemic country during the past 12 months, or planned travel to a malaria-endemic country during the course of the study; and receipt of chloroquine or other aminoquinolines within 12 weeks of study entry. Female volunteers were required to have a negative urine pregnancy test at least three days prior to vaccination and on the day of vaccination and to agree to use contraception or abstain from sexual intercourse for the duration of the study.

### Interventions

The MSP1_42_-C1 vaccine contains an equal mixture of two highly-purified, recombinant malaria proteins that correspond to the external domain of MSP1_42_ of the *P. falciparum* FVO and 3D7 strains. The recombinant proteins were each expressed in *E. coli* and purified by a combination of metal affinity chromatography, protein refolding and subsequent anion-exchange and size-exclusion chromatography as previously described [10]. Both protein drug substances were manufactured under current Good Manufacturing Practices (cGMP) at the Walter Reed Army Institute of Research, Pilot Bioproduction Facility (Silver Spring, MD). Equal weight amounts of the MSP1_42_-FVO and MSP1_42_-3D7 drug substances were mixed together and bound to aluminum hydroxide (Alhydrogel®, Brenntag Biosector A/S, Denmark). This formulation was performed under cGMP by the Pharmaceutical Development Section, Pharmacy Department, Clinical Center, National Institutes of Health (Bethesda, MD). The vaccine was supplied in single dose vials as a cloudy liquid containing MSP1_42_-C1 adsorbed to Alhydrogel®, in sterile saline solution (0.9% sodium chloride) without stabilizers or preservatives, for intramuscular (IM) injection. Two lots of the MSP1_42_-C1/Alhydrogel® vaccine were supplied to the clinical trial site, containing 40 or 160 µg of MSP1_42_-C1 and 404 µg of aluminum per 0.5 mL dose in 0.7 mL vials. Each vaccine lot underwent comprehensive quality control analysis to ensure that the purity, identity, and integrity of the product met specifications. Storage of the vaccine lots at the clinical site was confirmed to be between 2–8°C by a continuous temperature monitoring system (Rees Scientific, Trenton, NJ). The potency and stability of the vaccine lots were confirmed by evaluation of their immunogenicity in mice conducted every six months throughout the course of the trial.

CPG 7909 (Coley Pharmaceutical Group, Wellesley, MA) is a synthetic oligodeoxynucleotide and toll-like receptor 9 agonist that has been used as an adjuvant for vaccines targeting infectious diseases and cancer [13], [14]. CPG 7909 was manufactured according to cGMP standards and was supplied in sterile vials at 10 mg/mL in hypertonic phosphate buffered saline. CPG was added to MSP1_42_-C1/Alhydrogel at the point of injection shortly before vaccination, as previously described [11], to give a dose of 560 µg.

### Randomization and Blinding

Randomization was performed by the study pharmacist, who prepared the vaccines and kept the study code. The delivery volumes of the vaccines with and without CPG 7909 were slightly different (0.55 and 0.50 mL respectively), so opaque tape was used to mask the contents and maintain blinding by the person administering vaccines. The study was partially unblinded to assess outcomes after the third vaccinations; however, all clinical and immunological assessments were conducted by investigators who remained blinded until completion of the study.

### Outcomes

#### (i) Safety

The primary objective of the trial was to assess the safety of the MSP1_42_-C1/Alhydrogel® vaccine with and without CPG 7909. Following each vaccination, volunteers were observed for 60 minutes and then returned to the clinic on days 1, 3, 7, and 14 following vaccination for evaluation of local and systemic reactogenicity. Local reactogenicity was defined as erythema, swelling, induration, and pain at the injection site. Solicited systemic adverse events included fever (>37.5°C), headache, nausea, malaise, myalgia, arthralgia, and urticaria. A complete blood count with differential and platelet count was tested at each vaccination and on day 3, 7, and 14 following each vaccination. Serum alanine aminotransferase (ALT) and creatinine were tested at each vaccination and on days 3 and 14 following vaccination. Markers for autoimmunity (anti-double stranded DNA (dsDNA), anti-nuclear antibody (ANA), complement levels, and rheumatoid factor) were tested at study Day 0; anti-ds DNA was repeated on each vaccination day, 7 days after each vaccination, and 1 month and 6 months after the last vaccination. Urine for blood and protein were also tested as markers of autoimmunity on the day of and 14 days after each vaccination. Volunteers were given a digital thermometer, injection site reaction measurement tool, and symptom diary card to record their daily temperature, injection site reactions, and solicited adverse events for 14 days following each vaccination. All adverse events were graded for severity: mild (easily tolerated), moderate (interferes with daily activity or required remedial therapy), or severe (prevents daily activity). Injection site erythema, swelling, and induration were graded for severity as follows: mild (>0 to ≤20 mm), moderate (>20 mm to ≤50 mm), severe (>50 mm). The association of the adverse event with receipt of study vaccine was determined to be definite, probable, possible, unlikely, or unrelated.

#### (ii) Immunological assays

Measurements of anti-MSP1_42_ antibody levels were done on serum collected from participants on study Days 0, 14, 28, 42, 56, 70, 84, 140, and 238. The standardized methodology for performing the ELISA has been described previously [15] and OD-based ELISA units were converted to µg/mL as described previously [16].

The ability of induced anti-MSP1_42_ antibodies to inhibit growth of *P. falciparum* FVO and 3D7 parasites was assessed by a standardized growth inhibition assay (GIA) [17] using IgGs purified from study Days 0 and 70 sera. Briefly, each test IgG (10 mg/mL in a final test well) was incubated with synchronized *P. falciparum* parasites for ∼40 h and relative parasitemia levels were quantified by biochemical determination of parasite lactate dehydrogenase.

### Statistical Analysis

To compare the frequency of adverse events in the low versus high dose groups and non-CPG versus CPG groups, logistic regressions were performed for local and solicited events and for specific events for which 5 or more subjects had an occurrence. Exact logistic regression was used if no maximum likelihood estimate existed. Severity of local and solicited adverse events was compared using cumulative logit models with main effects for dose group and CPG, with each participant scored for maximum severity events as follows: severity = 0 (no AE observed), severity = 1 (mild), severity = 2 (moderate) and severity = 3 (severe). All subjects receiving any vaccinations were included in the analysis. No corrections were made for multiple analyses.

All data available for each time point for each subject were used for the immunological analysis, but for the graphical representation of antibody levels over time only subjects who did not miss any antibody measurements were included. Since there was a high level of concordance between the anti-MSP1_42_-FVO and -3D7 responses (data not shown), the arithmetic average of the response to both alleles was used as the response for each subject at each time point. Exact stratified Wilcoxon rank sum tests were used to calculate two-sided p-values for the effect on antibody responses of adding CPG, and to compare the dose groups. Tests were performed at both Day 42 and Day 70 (two weeks after the second and third vaccinations respectively), and at Day 236 (end of study). Fold-increases and confidence intervals were estimated using the Hodge-Lehmann method [18]. The correlation between antibody level and growth-inhibitory activity was tested by a Spearman rank test. For growth-inhibitory activity, the effect of adding CPG was tested by exact stratified Wilcoxon rank sum tests using Day 70 data. Comparison of GIA data between Day 0 and Day 70 was performed by the Wilcoxon signed rank test for each group. Calculations were done using R Version 2.9.1, SAS Version 9.1 or StatXact Procs Version 8.0 and p<0.05 was considered to be significant.

A group size of 15 participants gave a power per group of 0.8 for detecting serious or medically significant adverse events that occurred with a frequency of 0.1 per participant. A group size of 30 participants (in the non-CPG and CPG groups combined) gave a power of 0.8 for detecting a 2.4 fold difference in antibody responses between the groups, assuming a coefficient of variation in antibody responses of 1.7 and a significance level of 0.05.

## Results

### Participant Flow (Figure 1)

Sixty healthy adult volunteers (25 female and 35 male) were enrolled in the study. The mean age of volunteers was 30.5 years (range 18 – 50). Thirty volunteers were randomized 1∶1 to receive 40 µg MSP1_42_/Alhydrogel® (Group A) or 40 µg MSP1_42_/Alhydrogel® with CPG 7909 (Group B). An additional thirty volunteers were randomized 1∶1 to receive 160 µg MSP1_42_/Alhydrogel® (Group C) or 160 µg MSP1_42_/Alhydrogel® with CPG (Group D). Vaccinations began in March, 2006 and all follow up was complete by April, 2007. Fifty-one volunteers received all three vaccinations as scheduled. Five volunteers received only one vaccination and four volunteers received only two vaccinations; one volunteer was withdrawn after third vaccination. Of the five volunteers who received only one vaccination, one volunteer in Group A withdrew consent following the first vaccination, one volunteer in Group B was lost to follow-up prior to second vaccination, and three volunteers were medically ineligible to receive second vaccination due to an elevated ANA on day 0 prior to first vaccination (one volunteer each in Groups C and D; blood was drawn prior to vaccination but results were not available until after enrollment) or due to receipt of steroids prior to second vaccination (one volunteer in Group D). Of the four volunteers who did not receive a third vaccination, three did not receive a vaccination because they were unavailable for vaccination within the protocol-defined window (one volunteer each in Groups A, C, and D). The fourth volunteer did not receive a third vaccination due to receipt of steroids prior to third vaccination (Group C). One volunteer in Group D was withdrawn after third vaccination due to incarceration. Eight volunteers completed all vaccinations but missed one or more study visits and were missing data points for the graphical representation of the longitudinal ELISA antibody levels (1 in Group A, 2 in Group B, 2 in Group C, and 3 in Group D).

**Figure 1:**
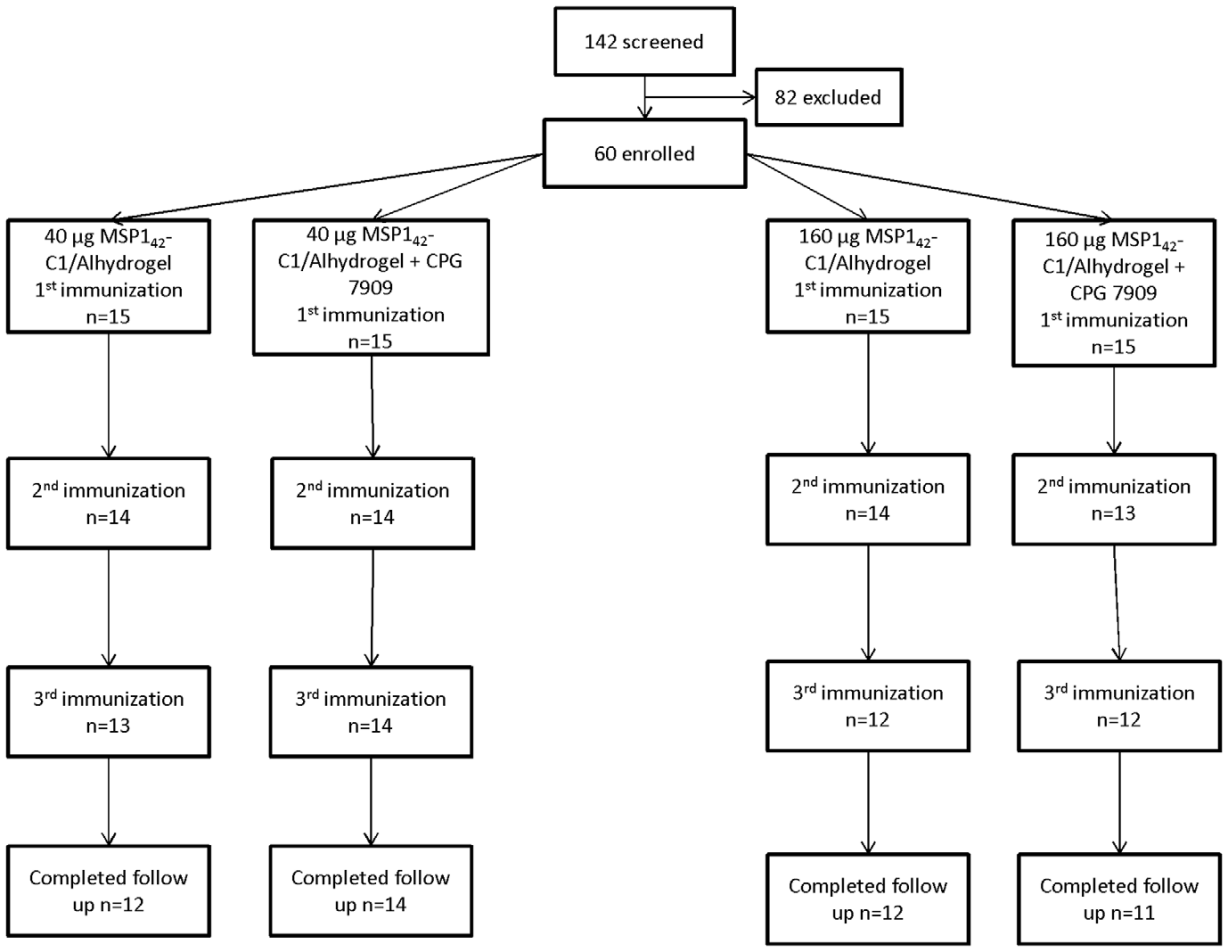
Flow chart of vaccinations and volunteer participation.

### Safety and Reactogenicity

No serious adverse events occurred during the study and no volunteers were withdrawn due to adverse events. Local reactogenicity, solicited adverse events and selected laboratory results are shown in **Tables 1** and **2**. All local adverse events were mild or moderate with the exception of severe erythema in one volunteer in Group D (high dose with CPG). Injection site pain was the most frequent injection site reaction and did not increase in frequency or severity with subsequent vaccinations. The odds of having any local adverse event were significantly greater in the CPG than in the non-CPG groups (p = 0.024) but there was no significant difference between the high and low dose groups (p = 0.65). The odds of having a more severe local adverse event was greater in the high dose than in the low dose groups (odds ratio (OR) = 6.8, 95% CI 1.7, 27.8, p = 0.008), and in the CPG groups compared to the non-CPG groups (OR = 23.6, 95% CI 2.9, 192.7, p = 0.003). All solicited adverse events were either mild or moderate and headache was the most frequent systemic adverse event overall. Solicited adverse events were not significantly more frequent (p = 0.30) nor more severe (OR = 1.6, 95% CI 0.6, 4.4, p = 0.4) in the high dose compared to the low dose groups. Similarly there was no significant difference between the CPG and the non-CPG groups in either the frequency (p = 0.30) or the severity (OR = 2.4, 95% CI 0.8, 7.1, p = 0.1) of solicited adverse events. Of the specific adverse events that occurred frequently enough to be included in the analysis, injection site swelling and malaise were more frequent in the CPG groups (p = 0.037 and 0.050 respectively) compared to the non-CPG groups. Upper respiratory infection (URI) was more frequent in the high dose groups (p = 0.014), but this is likely to be a statistical artifact as URI would not be expected to be related to vaccination. As expected, transient neutropenia was more frequent in the CPG groups (p = 0.014), as described below. No rashes occurred that were judged to be related to vaccination.

**Table 1:** Local adverse events.

	Vaccination 1				Vaccination 2				Vaccination 3			
	Total	Mild	Moderate	Severe	Total	Mild	Moderate	Severe	Total	Mild	Moderate	Severe
**40 µg MSP1_42_-C1/Alhydrogel®**												
Pain	9/15	9	0	0	10/14	10	0	0	3/13	3	0	0
Erythema	1/15	1	0	0	1/14	1	0	0	3/13	3	0	0
Swelling	0/15	0	0	0	0/14	0	0	0	1/13	1	0	0
Induration	0/15	0	0	0	0/14	0	0	0	0/13	0	0	0
**40 µg MSP1_42_-C1/Alhydrogel®+CPG 7909**												
Pain	11/15	11	0	0	10/14	9	1	0	9/14	9	0	0
Erythema	1/15	1	0	0	2/14	2	0	0	3/14	3	0	0
Swelling	1/15	1	0	0	0/14	0	0	0	2/14	2	0	0
Induration	1/15	1	0	0	0/14	0	0	0	0/14	0	0	0
**160 µg MSP1_42_-C1/Alhydrogel®**												
Pain	10/15	10	0	0	9/14	8	1	0	5/12	5	0	0
Erythema	1/15	1	0	0	1/14	1	0	0	2/12	2	0	0
Swelling	0/15	0	0	0	1/14	1	0	0	1/12	1	0	0
Induration	0/15	0	0	0	0/14		0	0	2/12	2	0	0
**160 µg MSP1_42_-C1/Alhydrogel®+CPG 7909**												
Pain	14/15	11	3	0	11/13	7	4	0	9/12	8	1	0
Erythema	0/15	0	0	0	2/13	2	0	0	1/12	0	0	1
Swelling	5/15	2	3	0	3/13	2	1	0	1/12	1	0	0
Induration	0/15	0	0	0	1/13	1	0	0	1/12	1	0	0

**Table 2:** Solicited systemic and selected laboratory adverse events.

Solicited AE	Vaccination 1				Vaccination 2				Vaccination 3			
	Total	Mild	Moderate	Severe	Total	Mild	Moderate	Severe	Total	Mild	Moderate	Severe
**40 µg MSP1_42_-C1/Alhydrogel®**												
Fever	0/15	0	0	0	0/14	0	0	0	0/13	0	0	0
Headache	4/15	4	0	0	1/14	1	0	0	3/13	3	0	0
Nausea	1/15	1	0	0	1/14	1	0	0	1/13	1	0	0
Malaise	3/15	3	0	0	0/14	0	0	0	0/13	0	0	0
Myalgia	1/15	1	0	0	0/14	0	0	0	0/13	0	0	0
Arthralgia	1/15	1	0	0	0/14	0	0	0	0/13	0	0	0
Leukopenia	2/15	2	0	0	2/14	2	0	0	0/13	0	0	0
Neutropenia	1/15	1	0	0	1/14	1	0	0	1/13	1	0	0
Anemia	0/15	0	0	0	0/14	0	0	0	0/13	0	0	0
Thrombocytopenia	0/15	0	0	0	0/14	0	0	0	0/13	0	0	0
Elevated ALT	0/15	0	0	0	0/14	0	0	0	1/13	1	0	0
**40 µg MSP1_42_-C1/Alhydrogel®+CPG 7909**												
Fever	1/15	1	0	0	2/14	2	0	0	1/14	1	0	0
Headache	6/15	5	1	0	7/14	6	1	0	3/14	3	0	0
Nausea	1/15	1	0	0	0/14	0	0	0	1/14	1	0	0
Malaise	3/15	2	1	0	2/14	2	0	0	1/14	1	0	0
Myalgia	3/15	3	0	0	2/14	2	0	0	1/14	1	0	0
Arthralgia	3/15	3	0	0	1/14	1	0	0	3/14	2	1	0
Leukopenia	2/15	2	0	0	1/14	1	0	0	1/14	1	0	0
Neutropenia	3/15	1	2	0	5/14	4	1	0	3/14	2	1	0
Anemia	0/15	0	0	0	0/14	0	0	0	0/14	0	0	0
Thrombocytopenia	0/15	0	0	0	0/14	0	0	0	0/14	0	0	0
Elevated ALT	0/15	0	0	0	2/14	2	0	0	0/14	0	0	0
**160 µg MSP1_42_-C1/Alhydrogel®**												
Fever	0/15	0	0	0	1/14	0	1	0	0/12	0	0	0
Headache	3/15	3	0	0	4/14	4	0	0	1/12	1	0	0
Nausea	2/15	2	0	0	1/14	1	0	0	0/12	0	0	0
Malaise	2/15	2	0	0	2/14	2	0	0	0/12	0	0	0
Myalgia	1/15	1	0	0	1/14	1	0	0	0/12	0	0	0
Arthralgia	0/15	0	0	0	0/14	0	0	0	0/12	0	0	0
Leukopenia	1/15	1	0	0	1/14	1	0	0	0/12	0	0	0
Neutropenia	0/15	0	0	0	0/14	0	0	0	0/12	0	0	0
Anemia	0/15	0	0	0	1/14	1	0	0	1/12	1	0	0
Thrombocytopenia	0/15	0	0	0	0/14	0	0	0	0/12	0	0	0
Elevated ALT	1/15	1	0	0	2/14	2	0	0	0/12	0	0	0
**160 µg MSP1_42_-C1/Alhydrogel®+CPG 7909**												
Fever	1/15	1	0	0	3/13	3	0	0	1/12	0	1	0
Headache	5/15	4	1	0	3/13	3	0	0	4/12	4	0	0
Nausea	5/15	5	0	0	1/13	1	0	0	2/12	2	0	0
Malaise	3/15	2	1	0	4/13	2	2	0	1/12	0	1	0
Myalgia	2/15	1	1	0	1/13	0	1	0	0/12	0	0	0
Arthralgia	2/15	1	1	0	0/13	0	0	0	0/12	0	0	0
Leukopenia	1/15	1	0	0	1/13	1	0	0	1/12	1	0	0
Neutropenia	2/15	2	0	0	2/13	2	0	0	0/12	0	0	0
Anemia	0/15	0	0	0	0/13	0	0	0	1/12	1	0	0
Thrombocytopenia	0/15	0	0	0	1/13	0	1	0	1/12	0	1	0
Elevated ALT	1/15	1	0	0	0/13	0	0	0	0/12	0	0	0

Transient neutropenia developed in 9 volunteers receiving the vaccine with CPG 7909: 2 individuals had moderate neutropenia (moderate neutropenia was defined as absolute neutrophil count between 500 and 999 cells/mm^3^; the lowest levels detected in the two volunteers were 626 and 961) and the others were mild (between 1000 and 1499 cells/mm^3^). Some volunteers had neutropenia after more than 1 vaccination but neutropenia did not become more severe with successive vaccinations. Neutropenia was also observed in one volunteer that received the vaccine without CPG. Neutropenia resolved by Day 7 post-vaccination for all volunteers. No clinical events were associated with the neutropenia. One volunteer in Group C developed a grade 1 leukocytosis after second vaccination and two volunteers in Group D developed a grade one leukocytosis after third vaccination; all resolved within 15 days. One volunteer in Group D developed a grade 1 leukocytosis 20 days after first vaccination; this was deemed remotely related to vaccine. Seven volunteers experienced mild elevation in serum alanine transaminase (ALT): 2 received the low dose vaccine with CPG, 1 received the low dose vaccine without CPG, 1 received the high dose vaccine with CPG, and 3 received the high dose vaccine without CPG. Four volunteers experienced elevation within three days of vaccination which lasted 4 to 12 days and was considered possibly related to vaccination; one volunteer experienced mild ALT elevation 13 days post-vaccination lasting 9 days and judged possibly related to vaccine; one volunteer experienced ALT elevation 28 and 115 days post-vaccination deemed remotely related to vaccination; and one volunteer experienced ALT elevation 20 days post-vaccination 1 that was deemed remotely related to vaccine. Two volunteers had elevated ANA (>1∶80) at enrollment and were not revaccinated as per protocol; 28 volunteers had low ANAs (1∶40) at enrollment and were continued in the study without the subsequent occurrence of events that posed risks to safety. No positive anti-dsDNA results occurred and no volunteer had a clinical event related to autoimmunity.

### Immune Responses

**Figure 2:**
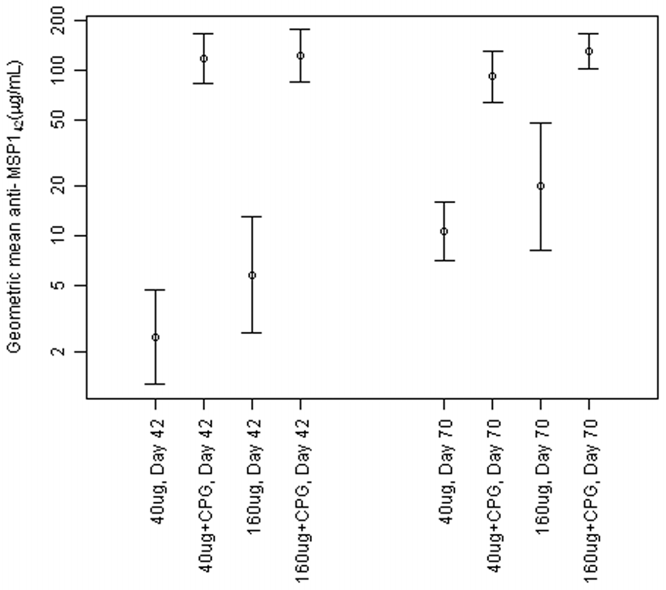
Geometric means and 95% confidence intervals of antibody in each group, as measured by ELISA and calculated from the arithmetic mean of anti-MSP1_42_-FVO and anti-MSP1_42_-3D7 ELISA results.

**Figure 3:**
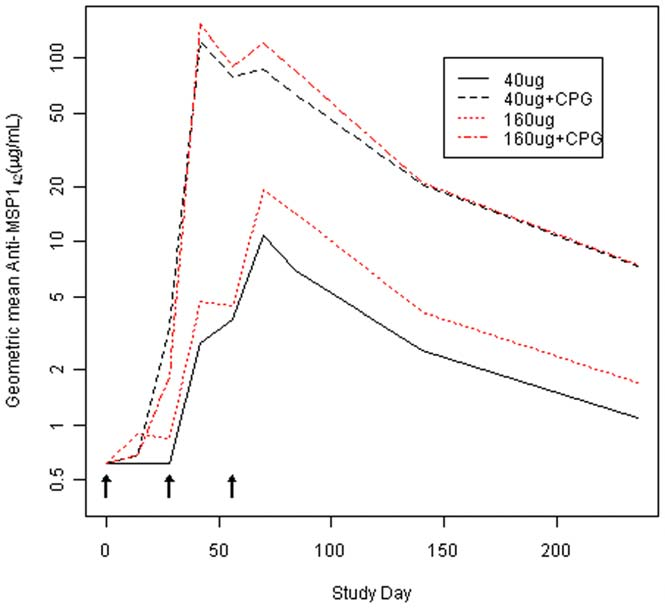
Geometric mean of average of FVO and 3D7 anti-MSP1_42_ (ug/mL) over time. Includes only subjects with samples at all time points (40 ug: n = 12, 40 ug+CPG: n = 12, 160 ug: n = 10, 160 ug+CPG: n = 8).

Geometric mean antibody responses after second and third immunizations are shown in **Figure 2**. The highest responses were in the groups that received 40 and 160 µg MSP1_42_/Alhydrogel with CPG at Day 42 (2 weeks after second vaccination), with geometric mean IgG concentrations of 118 and 121 µg/mL, respectively. The addition of CPG significantly enhanced antibody responses in both the 40 and 160 µg dose groups, at both Day 42 and Day 70, with the magnitude of the enhancement greater at Day 42 (after the second immunization), and for the 40 µg dose group (**Table 3**, all p values<0.0001). The greatest enhancement was seen at Day 42 in the low (40 µg) dose group, where a 49.4 fold increase was seen when CPG was added (95% CI 24.2–103.8). Antibody responses were not significantly higher in the 160 µg dose groups when compared to the corresponding low dose group with or without CPG, although there was a trend towards a higher response in the 160 µg without CPG group compared to the 40 µg without CPG group at day 70 (2.4 fold increase, 95% CI 0.9, 4.4, p = 0.07). Responses over time are shown in **Figure 3**. Antibody responses in the groups receiving CPG peaked after second vaccination, while those without CPG peaked after third vaccination. Antibody levels declined at similar rates in both dose groups, but remained significantly higher at Day 238 in the groups receiving CPG.

**Table 3:** Fold increase in antibody responses induced by vaccine with CPG 7909 compared to vaccine without CPG.

Dose Group and Study Day	Fold difference in FVO/3D7 average antibody response (95% Confidence Interval)
40 µg Day 42	49.4 (24.2, 103.8)
40 µg Day 70	8.1 (4.6, 13.6)
160 µg Day 42	20.7 (5.3, 60.4)
160 µg Day 70	4.81 (2.7, 12.4)

p<0.0001 for all tests.

**Figure 4:**
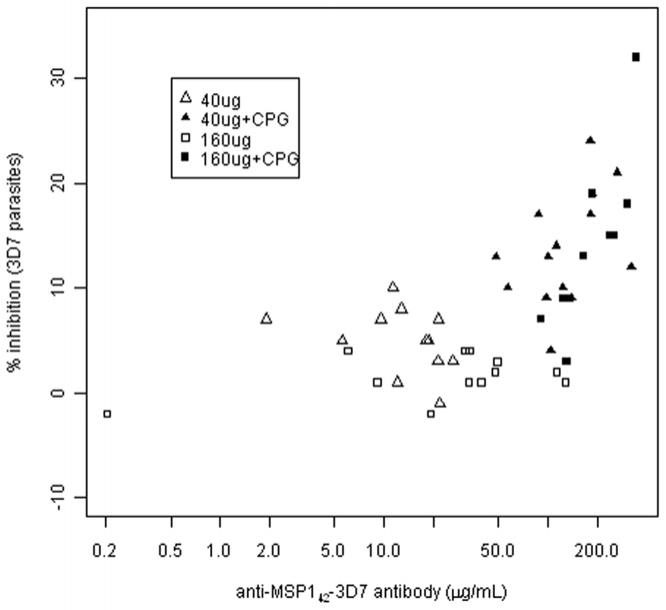
In vitro growth inhibition levels plotted against anti-MSP1_42_-3D7 antibody for 3D7 homologous parasites.

*In vitro* growth-inhibitory activity was assessed with individual IgGs from Day 0 and 70 sera using 3D7 parasites. While the average % inhibition of Day 0 IgGs was 1% for 3D7 parasites (ranging from −8 to 12%), Day 70 IgGs showed an average of 3% (ranging from −2 to 10%) in the non-CPG groups and 14% (3 to 32%) in the CPG groups. There was a small, but significant, increase in growth-inhibition activity in both non-CPG (Wilcoxon signed rank test, p = 0.0007) and CPG (p<0.0001) groups on Day 70 compared to Day 0. The Day 70 data are shown in **Figure 4**. Growth-inhibitory activity was correlated with ELISA antibody levels (Spearman correlation 0.66, 95% CI [0.46,0.79], p<0.0001). Groups with CPG added had significantly higher GIA at Day 70 than the groups without CPG added (p<0.0001). GIA results using FVO parasites were similar (data not shown).

## Discussion

Both vaccines investigated in this study were well tolerated, although local adverse events were more frequent and severe when CPG 7909 was added and more severe when higher doses were given. Neutropenia and malaise was more frequent in the CPG groups. A previous study, in which a blood stage malaria vaccine (AMA1-C1/Alhydrogel) was given to malaria naïve adults with and without CPG 7909, showed an increase in local adverse events and greater severity of local and systemic events with CPG, and several volunteers were withdrawn due to adverse events [11]. However, a subsequent Phase 1 study in malaria naïve adults showed a more favorable adverse event profile, and in Malian adults minimal local reactogenicity and no related systemic reactogenicity was seen [12], [19]. In this study only one Grade 3 adverse event related to vaccination occurred (severe erythema), and no volunteers were withdrawn due to adverse events. Transient neutropenia occurred in the groups receiving CPG, typically 3 days after vaccination with a return to baseline levels by Day 7. Transient neutropenia has been observed in previous studies of CPG 7909 administration and is believed to be due to immune stimulation and white blood cell sequestration [12], [20]. As in previous studies, no clinical events occurred related to this neutropenia and its relevance as a safety signal in this population is doubtful.

Autoimmune disease is a theoretical concern with administration of CPG adjuvants. This concern has been heightened since the occurrence of Wegeners Granulomatosis (an autoimmune vasculitis affecting the lungs and kidneys) in a subject who received a recombinant hepatitis B vaccine adjuvanted with ISS, an oligonucleotide similar to CPG 7909 [21]. However, the causality of a single occurrence in an early phase clinical trial is difficult to assess, and vasculitis has also been previously reported with licensed hepatitis B vaccines [22]. In this study no volunteers developed laboratory markers of autoimmune disease, despite the pre-existence of low-positive ANA in some volunteers, and no clinical autoimmune events occurred. To date, including this study, a total of 111 volunteers have received malaria antigens adjuvanted with Alhydrogel and CPG 7909 [11], [12], [19]. While this number is far too small to evaluate the risk of autoimmune disease or other rare adverse events, the marked enhancement of antibody responses with CPG must also be considered when weighing potential risk and benefit, particularly for a vaccine intended to prevent morbidity and mortality due to *P. falciparum* malaria. The safety profile in the target population, malaria exposed children, is as yet unknown.

Antibody is thought to be the primary mechanism of protection against blood stages of malaria [4], [5]. A previous study of AMA1-C1/Alhydrogel with and without CPG 7909 showed up to 14-fold enhancement of antibody responses when CPG was added [11]. In this study the addition of CPG also markedly enhanced antibody responses, with a 49.4 fold enhancement seen after second vaccination with the 40 µg dose compared to the group not receiving CPG. Also as in the previous study with AMA1-C1/Alhydrogel, responses in the groups receiving CPG peaked after the second vaccination, with no additional increase after the third vaccination, and responses were similar in the high and low dose groups when CPG was added. Thus CPG causes a dose sparing effect, where responses after two doses with 40 µg antigen are higher than responses after three doses with 160 µg without CPG, and are similar to two doses with 160 µg antigen with CPG. This dose sparing effect is particularly important in a strategy where multiple antigens are combined, as may be required for an effective blood stage malaria vaccine. Faster induction of antibody responses is also an advantage, and has been seen when CPG is added to hepatitis B vaccine [23]. Significant enhancement of antigen specific memory B cell responses was seen after vaccination with malaria antigens with CPG, consistent with the marked enhancement in antibody responses [24].

While no assays have been validated as surrogates of protection for malaria vaccines, in vitro GIA has been utilized to measure the biologic activity of antibodies elicited in clinical trials of blood stage antigens [25]–[27]. In vitro growth inhibition of up to 96% has been demonstrated with AMA1 vaccines [11], [12], [28]. Despite the high levels of antibody induced here, comparable to those induced when CPG was added to AMA1-C1/Alhydrogel [11], [12], the maximum level of growth inhibition induced in any volunteer was only 32%. A previous study has shown that more anti-MSP1_42_ antibody is required to reach the same level of inhibition in GIA compared to anti-AMA1 antibody [16], thus although higher antibody (and correspondingly higher GIA) was induced with the addition of CPG, a level of inhibition likely to be biologically significant was not achieved in any volunteer. A recent study suggested that Fc receptor-mediated protection is important in MSP1-induced immunity [29]. This protective mechanism cannot be measured by GIA. Therefore, in the case of MSP1_42_-based vaccines investigators may need to consider other assay(s) to evaluate the biologic function of the induced antibody.

A Phase 2b study in Kenyan children of a monovalent MSP1_42_ vaccine adjuvanted with AS02 failed to show protection against clinical malaria [9]. Geometric mean antibody levels induced by vaccination in that population were ∼23 µg/mL, less than those induced in malaria naïve adults in this study. Strain-specific effects have been demonstrated in an *Aotus* model when animals received an MSP1_42_ vaccine with complete Freund's adjuvant [8], and may also have played a part in the lack of benefit demonstrated in Kenyan children. The same *Aotus* study showed no protection with an MSP1_42_ vaccine adjuvanted with AS02, but showed some protection with the same vaccine in a water-in-oil adjuvant (ISA 720), and the authors speculate that a Th1-biased adjuvant such as CPG could be added to induce additional protection.

It seems likely that a recombinant blood stage malaria vaccine will require multiple proteins, both to overcome diversity of the allelic antigens expressed by the parasites and to overcome diversity of responses in the target population. Which combination of antigens is most likely to be protective is as yet unknown, with the epidemiologic evidence conflicting [30]–[33]. Clinical development of blood stage vaccines is largely empiric, since correlates of protection are unknown. Vaccines which have been judged to be safe and adequately immunogenic have gone forward to Phase 2b trials in African children, but given the lack of protection demonstrated to date this approach may no longer be feasible. A human blood stage challenge using parasite-infected erythrocytes is under development and may prove useful in allowing down selection of blood stage vaccine candidates prior to large field trials [34], [35]. While further clinical development of MSP1_42_/Alhydrogel + CPG 7909 as a separate candidate vaccine is not currently anticipated, the good safety profile, high antibody responses, and biologic function of the induced antibody demonstrated in this trial provide a rationale for the inclusion of MSP1_42_/Alhydrogel + CPG 7909 as part of a combination blood stage vaccine, which is currently in early phase clinical development (ClinicalTrials.gov Identifier: NCT00889616). Demonstration of homologous protection in either a human challenge or non-human primate model (using adjuvants suitable for use in humans) would provide support for continued clinical development, although such models are as yet unvalidated with respect to their ability to predict protection in the target population. Demonstration of likely benefit may be of particular importance when novel adjuvants with a limited safety profile, such as CPG 7909, are part of the clinical formulation.

## Supporting Information

Checklist S1 CONSORT Checklist.
(0.19 MB DOC)

Protocol S1 Trial Protocol.
(0.43 MB PDF)
