# Supplementary material for: Phase 1 Trial of the Plasmodium falciparum Blood Stage Vaccine MSP142-C1/Alhydrogel with and without CPG 7909 in Malaria Naïve Adults
Source: PLoS One. 2010 Jan 22;5(1):e8787. doi: 10.1371/journal.pone.0008787 (PMC2809736; doi:10.1371/journal.pone.0008787)
Supplement: Protocol S1 — Trial Protocol. (0.43 MB PDF) [file pone.0008787.s002.pdf]

Phase 1 Study of the Safety and Immunogenicity of $MSP1_{42}$-C1/Alhydrogel® with and without CPG 7909, an Asexual Blood Stage Vaccine for *Plasmodium falciparum* Malaria

Version:	5.0 (April 24, 2008)
WIRB Protocol Number	20051029
NIAID Protocol Number:	05-I-0240
JHU Protocol Number:	CIR 212
FDA IND Number:	12418
Project Assurance:	FWA #00000287

Proposed start date:	March 2006
Proposed end date:	March 2007

<u>Sponsored by:</u>
Regulatory Compliance and Human Subjects Protection Branch (RCHSPB)
National Institute of Allergy and Infectious Diseases (NIAID)
National Institutes of Health (NIH)

<u>Corporate Support:</u>
Coley Pharmaceutical Group, Inc.
Wellesley, Massachusetts

Version 5.0: 24 April 2008
<u>Previous Versions</u>
Version 2.7, dated 23 May 2006
Version 2.8, dated 29 June 2006
Version 2.9, dated 4 August 2006
Version 3.0, dated 28 November 2006
Version 4.0, dated 8 June 2007

TEAM ROSTER

**Clinical Investigators:**	**Anna Durbin, MD (Principal Investigator)**<br>Clayton Harro, MD<br>Donna Shaffer, MS, RNCS, FNP<br>Johns Hopkins University<br>Bloomberg School of Public Health<br>Center for Immunization Research<br>624 N. Broadway<br>Baltimore, MD 21205<br>(410) 955-1622
**Scientific Investigators:**	Ruth Ellis, MD<br>Louis Miller, MD<br>Malaria Vaccine Development Branch
**NIAID Regulatory Affairs:**	John Tierney, RN<br>RCHSPB, OCR, OD<br>NIAID, NIH<br>6700 B Rockledge Drive<br>Bethesda, MD 20892<br>(301) 429-9267
**Medical Monitor:**	Wellington Sun, M.D.<br>Walter Reed Army Institute of Research
**Protocol Statistician:**	William Pan, DrPH<br>Johns Hopkins University<br>Bloomberg School of Public Health<br>Department of International Health<br>(410) 502-2141

PARTICIPATING SITES

**Clinical Trial Site:**	**Center for Immunization Research (CIR)**<br>Johns Hopkins University<br>Bloomberg School of Public Health<br>2112 F Street, NW<br>Suite 305<br>Washington DC 20037<br>202-223-6564
	**Center for Immunization Research**<br>Johns Hopkins University<br>Bloomberg School of Public Health<br>624 N. Broadway<br>Baltimore, MD 21205
**Immunology Laboratory:**	**Malaria Vaccine Development Branch (MVDB)**<br>National Institutes of Allergy and Infectious Diseases (NIAID)<br>5460 Fishers Lane, Twinbrook I<br>Rockville, MD 20852<br>(301) 435-3405
	Laboratory of Malaria and Vector Research (LMVR)<br>NIAID/NIH<br>12735 Twinbrook Parkway, Twinbrook 3<br>Rockville, Maryland 20852<br>(301) 496-3655
	Laboratory of Immunogenetics (LIG)<br>NIAID/NIH<br>12441 Parklawn Drive, Twinbrook II<br>Rockville, Maryland 20852<br>(301) 435-3405
	Fisher BioServices (sample processing & cryopreservation)<br>685 Lostrand Lane,<br>Rockville, MD 20850<br>(301) 309-3668
**Clinical Laboratory:**	**Quest Diagnostics**<br>1901 Sulphur Springs Road<br>Baltimore, MD 21227<br>(800) 368-2576
**Apheresis Site**	Department of Transfusion Medicine<br>Building 10 1C710, Clinical Center<br>National Institutes of Health<br>9000 Rockville Pike<br>Bethesda, Maryland 20892<br>(301) 496-1430

TABLE OF CONTENTS

1.0 INTRODUCTION 8
1.1 Background 8
1.2 Vaccine Description 9
1.2.1 $MSP1_{42}$-C1 Drug Substance 9
1.2.2 Alhydrogel® 9
1.2.3 CPG 7909 9
1.3 Vaccine Formulation 9
1.4 Preclinical Experience with $MSP1_{42}$-C1 Vaccines 10
1.5 Clinical Experience with $MSP1_{42}$ Vaccines 10
1.6 Clinical Experience with Aluminum-Based Adjuvants 10
1.7 Clinical experience with CPG 7909 (VaxImmune®): 11
1.8 Clinical Development Plan 11
1.9 Participation of Children 11
2.0 OBJECTIVES 12
2.1 Primary Objectives 12
2.2 Secondary Objectives 12
2.3 Tertiary Objectives – the following will be for information only 12
3.0 STUDY DESIGN 12
3.1 Overall design: 12
3.2 Sample Size and Estimated Duration of Study 14
3.3 Group Allocation 14
3.4 Blinding 14
4.0 SELECTION AND ENROLLMENT OF PARTICIPANTS 15
4.1 Inclusion criteria: 15
4.2 Exclusion criteria: 15
4.3 Treatments that could potentially interfere with vaccine-induced immunity: 16
4.4 Contraindications to vaccination: 16
4.5 Indications for deferral of vaccination: 16
4.6 Participant withdrawal criteria: 17
5.0 VACCINE PREPARATION 17
5.1 Supplies 17
5.1.1 $MSP1_{42}$-C1/Alhydrogel® 18
5.1.2 CPG 7909 18
5.2 Vaccine Storage 18
5.3 Vaccine Accountability 18
5.4 Disposition of Used/Unused Supplies 18
6.0 STUDY PROCEDURES 19
6.1 Screening (up to 60 days prior to vaccination): 19
6.2 Randomization Process 19
6.3 Screening Process and Enrollment 19
6.4 Immunization procedure: 20
6.5 Clinical monitoring and evaluation: 20
6.6 Participant symptom diary: 24
6.7 Laboratory testing: 24

6.8 Immunologic testing: 25
6.8.1 Antibody assay (ELISA): 25
6.8.2 In vitro Parasite Growth inhibition assay (GIA): 25
6.8.3 Assessment of cellular immune responses 26
6.9 Use, Storage, and Tracking of Specimens and Data 26
6.10 Retention of Specimens for Future Use 26
7.0 ADVERSE EVENTS MONITORING AND REPORTING 27
7.1 Definitions: 27
7.1.1 Adverse Event (AE): 27
7.1.2 Serious Adverse Event (SAE): 27
7.2 Assessment of adverse events: 27
7.2.1 Identification of adverse events: 27
7.2.2 Determination of severity: 28
7.2.3 Association with receipt of the study vaccine: 29
7.4 Adverse event reporting: 30
7.5 Adverse event monitoring: 31
7.5.1 Medical monitor: 31
7.5.2 Data Safety and Monitoring Board (DSMB): 31
7.6 Stopping Criteria: 32
7.7 Breaking the study blind 33
8.0 DATA COLLECTION AND MONITORING 33
8.1 Source Documentation: 33
8.2 Study Documentation: 33
8.3 Retention of Records: 34
8.4 Protocol Revisions: 34
8.5 Clinical Investigators' Brochure: 34
8.6 Study Monitoring: 34
9.0 STATISTICAL CONSIDERATIONS 35
9.1 Description of the statistical methods to be employed: 35
9.1.1 Safety 36
9.1.2 Immunogenicity Analysis 37
9.2 Sample Size 37
10.0 PROTECTION OF HUMAN SUBJECTS 37
10.1 Institutional Review Board/Ethics Committee 37
10.2 Informed Consent: 37
10.3 Risks: 38
10.3.1 Venipuncture 38
10.3.2 Immunization 38
10.4 Benefits 38
10.5 Confidentiality: 38
10.6 Compensation: 38
11.0 REFERENCES 39
APPENDIX A: SCHEDULE OF VISITS 41
APPENDIX B - TOXICITY TABLE FOR GRADING LABORATORY ADVERSE EVENTS 43

APPENDIX C – TOXICITY TABLE FOR GRADING UNEXPECTED SYSTEMIC ADVERSE EVENTS 44
APPENDIX D – MALARIA INFORMED CONSENT COMPREHENSION TEST 46

PROTOCOL SUMMARY

Protocol Title: Phase 1 Study of the Safety and Immunogenicity of MSP1-C1_{42}/Alhydrogel® +/- CPG 7909, an Asexual Blood Stage Vaccine for *Plasmodium falciparum* Malaria

Version: 5.0

Revision History: May 23, 2006, June 29, 2006, August 4, 2006, November 28, 2006, June 8, 2007, April 24, 2008

Participants: Healthy malaria-unexposed male and non-pregnant female participants aged 18-50 years

Number of Participants: n = 60

Trial Design: Double blind, randomized, Phase 1 dose escalating clinical trial

Immunization Schedule:

MSP1_{42}-C1/Alhydrogel®±CPG 7909 Vaccine Groups

Group	Number of Participants	Immunization Schedule		
		Day 0	Day 28	Day 56
A	15	A (15)	A (15)	A (15)
B	15	B (15)	B (15)	B (15)
C	15	C (15)	C (15)	C (15)
D	15	D (15)	D (15)	D (15)
Total	60	A: 40 µg MSP1_{42}-C1/Alhydrogel®<br>B: 40 µg MSP1_{42}-C1/Alhydrogel®+CPG7909<br>C: 160 µg MSP1_{42}-C1/Alhydrogel®<br>D: 160 µg MSP1_{42}-C1/Alhydrogel®+CPG7909		

Product Description: The vaccine preparations to be studied contain an equal mass mixture of MSP1_{42} from two different clones of *Plasmodium falciparum* (FVO and 3D7), each produced separately as recombinant proteins expressed by *Escherichia coli* (EcMSP1_{42}-FVO and EcMSP1_{42}-3D7). The MSP1_{42} drug substances were purified from solubilized inclusion bodies and the correctly folded material purified by a combination of metal affinity, anion-exchange and size-exclusion chromatography. Purified MSP1_{42}-FVO and MSP1_{42}-3D7 Drug Substances were subsequently mixed and adsorbed onto aluminum hydroxide gel (Alhydrogel®, Brenntag, Denmark) and mixed with CPG 7909.

Time Period: A total of approximately 53 weeks including vaccinations, study procedures, and follow-up of all participants. Each participant will be followed for a total of 34 weeks.

1.0 INTRODUCTION

1.1 Background

As reported by the World Health Organization in 2002, the worldwide incidence of malaria is approximately 300 million clinical cases annually, with approximately one million deaths per year attributed to malaria alone or in combination with other diseases [1]. Most of the mortality occurs among children under 5 years of age in sub-Saharan Africa. Of the four species of malaria that infect humans, *Plasmodium falciparum* is responsible for the majority of these deaths. Mounting drug resistance of the malaria parasite, as well as widespread resistance of mosquitoes to insecticides make these control strategies increasingly unrealistic. A vaccine that would reduce both mortality and morbidity secondary to *P. falciparum* infection would be a valuable resource in the fight against this disease.

Proteins expressed by *P. falciparum* are generally specific to one stage of the parasite's life cycle. Several *P. falciparum* merozoite antigens have been identified as potential blood-stage vaccine candidates [2]. Merozoite surface protein 1 (MSP1), the first protein to be identified on the surface of the blood-stage parasite [3], is synthesized as a ~200kDa polypeptide. MSP1 is processed at, or just prior to, merozoite release from the red blood cell into four smaller fragments, $MSP1_{83}$, $MSP1_{30}$, $MSP1_{38}$ and $MSP1_{42}$ which form a non-covalently associated complex [4]. The 42kDa fragment $MSP1_{42}$ is responsible for tethering the complex to the surface of the merozoite via a glycosylphosphatidylinositol (GPI) anchor [5,6]. At the time of merozoite invasion of erythrocytes, $MSP1_{42}$ undergoes a secondary processing event and is cleaved into $MSP1_{33}$ and $MSP1_{19}$ [7]. $MSP1_{19}$ is the only part of MSP1 carried into the newly invaded erythrocyte on the surface of the parasite [8].

The $MSP1_{42}$-FVO/Alhydrogel® and $MSP1_{42}$-3D7/Alhydrogel® vaccines have been tested individually in one human clinical trial. The trial started in July 2004 in Kansas. The vaccines were tested at 5, 20, and 80 µg doses at a 0, 28, 180 day schedule. The participants in each of the 3 dose groups have each received at least two vaccinations to date. The participants in the 5 µg dose groups have received three vaccinations. There have been no safety concerns related to vaccination in this study to date.

The development and testing of the individual vaccines, $MSP1_{42}$-FVO/Alhydrogel® and $MSP1_{42}$-3D7/Alhydrogel®, is a prelude to the use of these antigens in the combination vaccine, $MSP1_{42}$-C1/Alhydrogel® (1:1 mixture by mass of $MSP1_{42}$-FVO and $MSP1_{42}$-3D7 formulated on Alhydrogel®). The specificity of the antibody response to $MSP1_{42}$ following vaccination could have major implications on the use of a malaria vaccine. Vaccines that induce immunity against the most prevalent forms of the parasite would most effectively eliminate or reduce the emergence of breakthrough parasites in the immunized individual. The FVO and 3D7 forms of $MSP1_{42}$ cover both known dimorphisms in $MSP1_{33}$ and the major point mutations identified in $MSP1_{19}$. Therefore, a combination vaccine based on $MSP1_{42}$-FVO and $MSP1_{42}$-3D7 would address the concerns of targeting a polymorphic protein in generating protective immune responses.

CPG 7909 will be added to the MSP1-C1/Alhydrogel® formulation with the expectation that it will produce a higher level of antigen specific antibodies in humans, which is felt to be necessary for an effective malaria vaccine. Additionally, CPG 7909 is known to induce a Th1-biased immune response which has been shown to be more effective for inducing cell-mediated immune responses. CPG 7909 has been administered to humans on its own as an investigational

cancer therapeutic agent and as a vaccine adjuvant with two licensed vaccines to healthy adult participants without significant safety concerns.

1.2 Vaccine Description

1.2.1 $MSP1_{42}$-C1 Drug Substance

Both recombinant $MSP1_{42}$-FVO and $MSP1_{42}$-3D7 are highly purified proteins that correspond to the external domain of $MSP1_{42}$ of *P. falciparum* FVO and 3D7 lines, respectively. The proteins consist of the $MSP1_{42}$ fragment sequence excluding the glycosylphosphatidylinositol (GPI) anchor sequence. The $MSP1_{42}$-FVO and $MSP1_{42}$-3D7 Drug Substances each contain an additional 8 amino acids not found in the native $MSP1_{42}$ sequence; the addition of a Leu-Glu dipeptide sequence results from gene cloning and a 6-histidine C-terminal tag to allow for efficient purification of the protein. As *E. coli* codon usage is significantly different from *P. falciparum*, the native $MSP1_{42}$ nucleotide sequences were optimized for expression in *E. coli*. The synthetic $MSP1_{42}$-FVO and $MSP1_{42}$-3D7 sequences were subcloned into the expression plasmids, pET-24d(+) and pET-24a(+) respectively (Novagen). The recombinant proteins $MSP1_{42}$-FVO and $MSP1_{42}$-3D7 were each expressed in BL21(DE3) *E. coli* and purified from solubilized inclusion bodies by a combination of metal affinity chromatography, protein refolding and subsequent anion-exchange and size-exclusion chromatography. The purification process was designed to separate full-length, correctly folded product from degraded material as well as nonproduct related impurities. $MSP1_{42}$-FVO and $MSP1_{42}$-3D7 bulk Drug Substances were supplied in sterile phosphate buffered saline (pH 7.4) containing 0.2% Polysorbate 80 (Tween 80), as a stabilizer. Both Drug Substances were manufactured under cGMP conditions at the Walter Reed Army Institute of Research (WRAIR), Pilot Bioproduction Facility (Silver Spring, Maryland). Prior to release, the Drug Substances underwent comprehensive quality control analysis to ensure that product purity, identity and integrity met specifications.

1.2.2 Alhydrogel®

Aluminum hydroxide gel (Brenntag, Denmark) has been extensively used as an adjuvant in many licensed human vaccines. Aluminum-containing adjuvants are in routine human use and are contained in many licensed human vaccines. Alhydrogel® is supplied sterile, in isotonic saline without preservatives.

1.2.3 CPG 7909

CPG 7909 (Coley Pharmaceutical Group, Wellesley, MA) is a short synthetic oligodeoxynucleotide of the following sequence: 5'-TCG TCG TTT TGT CGT TTT GTC GTT-3', with all nucleotides linked with phosphorothioate bonds. CPG 7909 was manufactured according to cGMP standards and is supplied in sterile vials at 10 mg/mL in hypertonic buffer (154 mM NaCl, 94mM Na_2HPO_4, 6 mM NaH_2PO_4). VaxImmune™ (CPG 7909 Injection) is formulated as a sterile solution for adjunctive intramuscular administration.

1.3 Vaccine Formulation

$MSP1_{42}$-C1 refers to an equal mixture of $MSP1_{42}$-FVO and $MSP1_{42}$-3D7 based on weight. The $MSP1_{42}$-C1/Alhydrogel® vaccine refers to $MSP1_{42}$-C1 formulated on Alhydrogel®. The Alhydrogel® formulation consists of a total of 40 or 160 µg of $MSP1_{42}$-C1 and 404 µg of Aluminum per 0.5 mL dose and is supplied in vials containing 0.7 mL. Shortly before

vaccination, 0.08 mL of CPG 7909 will be mixed with 0.7 mL of $MSP1_{42}$-C1/Alhydrogel® to give a final injected CPG 7909 dose of 0.56 mg This formulation is referred to as $MSP1_{42}$-C1/Alhydrogel® + CPG 7909.

1.4 Preclinical Experience with $MSP1_{42}$-C1 Vaccines

A total of 9 individual preclinical trials have been conducted to assess the safety and immunogenicity of the $MSP1_{42}$-C1/Alhydrogel® +/- CPG 7909 vaccine prior to human clinical trials. Three of these studies have shown that the addition of CPG 7909 to $MSP1_{42}$/Alhydrogel® formulations improved the immune response. Two studies demonstrate stability and potency of the reference and clinical $MSP1_{42}$-C1/Alhydrogel® formulations. The remaining 4 studies established the rationale, safety and immunogenicity of $MSP1_{42}$-C1/Alhydrogel®.

$MSP1_{42}$-C1/Alhydrogel® + CPG 7909 has been tested by intramuscular (IM) administration in mice and rats, and by subcutaneous (SC) administration in mice. The $MSP1_{42}$-C1/Alhydrogel® + CPG 7909 formulation was more immunogenic in the animals as compared to $MSP1_{42}$-C1/Alhydrogel® given without CPG 7909. No clinically significant adverse effects due to vaccination have been reported.

1.5 Clinical Experience with $MSP1_{42}$ Vaccines

The vaccine formulations in this protocol have not been tested in human trials. There has been one human clinical trial to date using the individual recombinant $MSP1_{42}$-FVO/Alhydrogel® and $MSP1_{42}$-3D7/Alhydrogel® vaccines. Drug Substances comparable to those used in the clinical testing of the individual $MSP1_{42}$ forms have been used to generate the $MSP1_{42}$-C1/Alhydrogel® vaccine.

The trial of the $MSP1_{42}$-FVO/Alhydrogel® and $MSP1_{42}$-3D7/Alhydrogel® vaccines is being performed at Quintiles Phase 1 Services (Lenexa, KS). The trial began in July 2004. Sixty participants are scheduled to receive 3 immunizations (Days 0, 28 and 180) of either 5, 20 or 80 µg doses of either $MSP1_{42}$-FVO/Alhydrogel® OR $MSP1_{42}$-3D7/Alhydrogel® vaccines in an open label dose escalating study. To date, the 5 µg groups have received three vaccinations and the 20 µg and the 80 µg dose groups have received two vaccinations. There have been no safety concerns to date. Adverse events related to vaccination have been primarily local injection site reactions, which have been mild to moderate in severity.

1.6 Clinical Experience with Aluminum-Based Adjuvants

Several licensed vaccines contain aluminum-based adjuvants, including the recombinant Hepatitis B vaccine (Recombivax HB®) and the diphtheria-tetanus toxoids vaccine (DT) [9,10]. Recombivax HB® may be a particularly useful comparator vaccine, as it consists of a recombinant protein expressed in *Saccharomyces cerevisiae* and is administered IM. For these two aluminum-adsorbed vaccines, local reactions such as pain, tenderness, and swelling are experienced in between 7.6% and 16.7% of participants in studies that included over 1,200 healthy adults. Fever is seen in 3.2% to 9.3%, headache in 4.1%, and other systemic symptoms such as fatigue, malaise, nausea, and diarrhea at lower frequencies. Urticaria has been reported in 0.1% of individuals vaccinated with Recombivax HB®. This data is based on the Recombivax HB® vaccine that also contained the preservative thimerosal, which may increase reactogenicity.

1.7 Clinical experience with CPG 7909 (VaxImmune®):

This CPG motif has been administered to humans in combination with the Engerix-B® Hepatitis B vaccine and the Fluarix® killed influenza vaccine. Initial results from these two Phase 1 clinical trials indicate that the addition of CPG 7909 to these two licensed vaccines was safe, and in the case of Engerix-B®, induces significantly earlier and stronger antibody responses than the vaccine alone [11,12].

The Engerix-B® trial was a randomized, double-blind study in 56 healthy volunteers in Canada. This was a dose escalating study comparing 0.125, 0.5, and 1.0 mg of CPG 7909 admixed with the licensed Engerix-B® vaccine. The most frequently reported adverse events were injection site reactions (pain and erythema), flu-like symptoms and headache. All adverse events were mild or moderate in severity. One participant experienced a hypersensitivity type reaction immediately following the third dose of Engerix-B® + 1.0 mg of CPG 7909. The symptoms included warmth, weakness, nausea, and dizziness. The symptoms resolved without treatment. Additionally, one participant who received Engerix-B® + 1.0 mg of CPG 7909 had periodic elevations in anti-dsDNA which was initially detected two weeks after the second and third vaccinations. The anti-dsDNA returned to normal prior to receipt of the third dose and was normal at the end of the study. The participant was asymptomatic, and ANA and rheumatoid factor remained negative throughout.

The Fluarix® trial was a randomized, controlled, double-blind study in 60 healthy participants in Canada. Participants received either the licensed Fluarix® vaccine with or without CPG 7909, or 1/10th the dose of Fluarix® with or without CPG 7909. The most frequently reported adverse events were injection site pain, headache, myalgia and fatigue. All injection site reactions were mild or moderate in severity, with the exception of one participant in the 1/10th dose Fluarix® + CPG 7909 group who reported severe pain which resolved without treatment within four days. Transient reductions in total WBC, neutrophils, lymphocytes, eosinophils and platelets were observed in all four study arms 2 days post-vaccination. None of these results were felt to be clinically significant.

1.8 Clinical Development Plan

Assuming no significant safety issues are identified in this initial Phase 1 trial, we plan to proceed to a malaria-endemic area for Phase 1 and 2 trials with the $MSP1_{42}$-C1/Alhydrogel® + CPG 7909 formulation. A Phase 1 trial of this formulation will be repeated in healthy, malaria-exposed adults in a malaria-endemic region, given the possibility that the safety of this vaccine formulation may be different in such a population. Provided there are no safety concerns, age de-escalation, Phase 2, and eventually Phase 3 clinical trials will be undertaken in malaria-endemic areas.

1.9 Participation of Children

The vaccine formulation being tested in this protocol has not yet been tested in humans. It is felt that insufficient data are available to judge the potential risk in children. Once safety is established in adults in the United States and then in an endemic region, we plan to age de-escalate to children in malaria endemic regions.

2.0 OBJECTIVES

2.1 Primary Objectives

1. To assess the safety, reactogenicity and immunogenicity of the $MSP1_{42}$-C1/Alhydrogel® with and without CPG 7909.

2.2 Secondary Objectives

1. To demonstrate that the addition of CPG 7909 improves the specific immune responses to $MSP1_{42}$-FVO and $MSP1_{42}$-3D7, as compared to $MSP1_{42}$-C1/Alhydrogel® at day 70.
2. To determine the dose of $MSP1_{42}$-C1/Alhydrogel® + CPG 7909 that generates the highest serum antibody levels of $MSP1_{42}$-FVO and $MSP1_{42}$-3D7 at day 70.

2.3 Tertiary Objectives – the following will be for information only

1. To measure the biological activity of the antisera by an in vitro parasite growth inhibition assay using FVO and 3D7 parasites.
2. To determine the fine specificities and functionality of vaccine-induced antibody as judged by ELISA, GIA and IFA
3. To assess and compare the duration of antibody responses to $MSP1_{42}$-FVO and $MSP1_{42}$-3D7 proteins by ELISA over an 8 month period.
4. To perform exploratory studies of B and T cell populations both before and after vaccination.

3.0 STUDY DESIGN

3.1 Overall design:

The study is a randomized, double blind, Phase 1 clinical trial in healthy malaria-naïve adult participants designed to evaluate the safety, reactogenicity and immunogenicity of the $MSP1_{42}$-C1 malaria vaccine formulated on Alhydrogel® +/- CPG 7909. There will be 4 groups in this trial. Each group (A-D) will be comprised of 15 participants. Groups A and B will be enrolled and vaccinated simultaneously followed by Groups C and D. This study will be staggered such that dose escalation will not occur until the lower dose groups (A & B) have received 3 vaccinations at days 0, 28 and 56. Vaccination of each group of 15 volunteers will also be staggered such that 5 volunteers from Groups A and B will be vaccinated and at least one week later, the remaining 10 volunteers from groups A and B will be vaccinated. After all volunteers from groups A and B have been followed for at least 4 weeks after their third vaccination, 5 volunteers each from groups C and D will receive their first vaccination. At least one week later, the remaining 10 volunteers from groups C and D will receive their first vaccination.

There will be a total of 60 participants in this trial. Volunteers will be recruited from a variety of sources including: volunteers previously enrolled in vaccine trials at the CIR; by the use of a general screening protocol that has been in use at the CIR for more than 20 years and is approved by the Committee on Human Research, the Johns Hopkins Bloomberg School of Public Health IRB and by the use of study-specific IRB-approved print and/or other media advertising. Up to 600 participants may be screened, to allow for screening failures, with a goal of enrolling 15 participants into one of four groups, all of whom will receive three vaccinations. The first 30 participants will be randomized to receive either the 40 µg dose of $MSP1_{42}$-

C1/Alhydrogel® vaccine with or without CPG 7909. The next 30 participants will be randomized to receive either the 160 µg dose of $MSP1_{42}$-C1/Alhydrogel® vaccine with or without CPG 7909. (**See Table 6-1**).

After providing written informed consent, participants will undergo eligibility screening, including medical history, physical examination, hematology testing, liver and renal function testing, HIV, Hepatitis B and C screening, rheumatoid factor, anti-dsDNA and ANA testing, and urinalysis; pregnancy testing will be performed on female participants. For participants who are eligible, the Day 0 visit will be scheduled to receive the first dose of vaccine. Participants will be observed for immediate reactions following each vaccination for 60 minutes. Participants will return to the clinic on Days 1, 3, 7 and 14 following each vaccination for clinical assessment. See Table 6-1 for a tabular description of the vaccination schedule.

As with other aluminum hydroxide-adsorbed vaccines, hypersensitivity reactions would be expected to occur within the first 24 hours after receipt of the vaccine, and other severe local or systemic reactions within 72 hours of vaccination.

The cumulative safety data up to and including day 70 post-vaccination will be reviewed prior to dose escalation by the Data Safety Monitoring Board (DSMB). The trial will not proceed to the next cohort if, in the clinical judgment of the investigators or DSMB, the next higher dose would pose an unacceptable safety risk to the participants.

Table 6-1: Dose Escalation Schedule

**Time (Week, approximate [1])**	**Group A: (n=15)**<br><br>**40 µg $MSP1_{42}$/Alhydrogel® (n=15)**	**Group B: (n=15)**<br><br>**40 µg $MSP1_{42}$/Alhydrogel® + CPG 7909 (n=15)**	**Group C: (n=15)**<br><br>**160 µg $MSP1_{42}$/Alhydrogel® (n=15)**	**Group D: (n=15)**<br><br>**160 µg $MSP1_{42}$/Alhydrogel® + CPG 7909 (n=15)**
0	Vax 1a (day 0) n=5	Vax 1a (day 0) n=5		
1	Vax 1b (day 0) n=10	Vax 1b (day 0) n=10		
2, 3				
4	Vax 2a (day 28) n=5	Vax 2a (day 28) n=5		
5	Vax 2b (day 28) n=10	Vax 2b (day 28) n=10		
6, 7				
8	Vax 3a (day 56) n=5	Vax 3a (day 56) n=5		
9	Vax 3b (day 56) n=10	Vax 3b (day 56) n=10		
10, 11, 12				
13			Vax 1a (day 0) n=5	Vax 1a (day 0) n=5
14			Vax 1b (day 0) n=10	Vax 1b (day 0) n=10
15, 16				
17			Vax 2a (day 28) n=5	Vax 2a (day 28) n=5
18			Vax 2b (day 28) n=10	Vax 2b (day 28) n=10
19, 20				
21			Vax 3a (day 56) n=5	Vax 3a (day 56) n=5
22			Vax 3b (day 56) n=10	Vax 3b (day 56) n=10

These times are approximate and represent the earliest dates vaccinations will occur.

3.2 Sample Size and Estimated Duration of Study

A total of 60 participants will be enrolled, but to ensure that we achieve this total, an accrual ceiling of 600 will be set to accommodate possible screening failures. Thirty participants will receive one of 2 doses of the $MSP1_{42}$/Alhydrogel® vaccine: 40 µg, comprising of 20 µg each of $MSP1_{42}$ 3D7 and $MSP1_{42}$ FVO, or 160 µg, comprising of 80 µg each of $MSP1_{42}$ 3D7 and $MSP1_{42}$ FVO. Another 30 participants will receive one of the same 2 doses of the $MSP1_{42}$/Alhydrogel® + CPG 7909 vaccine. The trial is expected to last for a total of approximately 53 weeks. Each participant will be followed for 34 weeks from the time of the first injection; 6 month follow-up from the 3rd vaccination.

3.3 Group Allocation

The low dose groups (A & B) will be enrolled and vaccinated simultaneously with the first 5 participants of each group receiving vaccine followed by the next 10 participants one week later. Groups C & D will be vaccinated in the same manner but will be staggered such that dose escalation will not occur until the lower dose groups (A & B) have received 3 vaccinations at days 0, 28 and 56 and have day 70 post-vaccination cumulative safety data reviewed.

Once screening has started, the first 30 eligible participants will be randomly assigned to Group A or Group B, and a Day 0 visit will be scheduled. We will ask 2-4 alternates to attend each of the first vaccination days to ensure the entire group will be filled with eligible participants. As explained in Section 3.1, the goal will to enroll a total of 15 participants in each of the four groups at the end of the enrollment period. If the alternates are not vaccinated, they will be invited to participate as members of the next dose groups. Groups C & D will be enrolled and allocated in the same manner as described above. Alternates will be asked to attend each of the first vaccination days for Groups C and D. Volunteers who attend as alternates on the day of first vaccination, but are not enrolled, will receive payments for the screening visit ($50) and for one clinic visit ($75). .

3.4 Blinding

The investigators and participants will be blinded as to an individual study participant's allocation to either $MSP1_{42}$/Alhydrogel® vs. $MSP1_{42}$/Alhydrogel® + CPG 7909. Only the study drug manager/pharmacist will be aware of this allocation. The pharmacist will refer to the unique randomization code assigned to that participant to determine the assigned vaccine for each participant. Vaccine will be prepared by the study pharmacist in a separate room from the vaccination rooms. Since $MSP1_{42}$/Alhydrogel® and $MSP1_{42}$/Alhydrogel® + CPG 7909 are of slightly different volumes (0.50 mL and 0.55 mL, respectively), the contents of the syringes will be disguised using opaque tape.

Due to the staggered, dose escalation design of the trial, it will not be possible to blind to the dose of $MSP1_{42}$ that an individual may have received. Since Groups A and B (40 µg $MSP1_{42}$/Alhydrogel® with or without CPG 7909) will be vaccinated before Groups C and D (160 µg $MSP1_{42}$/Alhydrogel® with or without CPG 7909), it will not be possible to blind to the dose of $MSP1_{42}$-C1, only to the allocation between CPG and no CPG. The study will be unblinded to non-clinical investigators after volunteers in groups C and D reach study day 70 to assess safety and immunogenicity. Clinical investigators and study subjects will remain blinded to treatment assignment until all subjects in groups C and D have reached study day 236.

4.0 SELECTION AND ENROLLMENT OF PARTICIPANTS

4.1 Inclusion criteria:

1. Age between 18 and 50 years, inclusive.
2. Good general health as a result of review of medical history and/or clinical tests at screening.
3. Available for the duration of the trial (34 weeks).
4. Willingness to participate in the study as evidenced by signing the informed consent document.

4.2 Exclusion criteria:

1. Pregnancy as determined by a positive urine or serum β-hCG (if female).
2. Participant unwilling to use reliable contraception methods for the duration of the trial.

   Reliable methods of birth control include: pharmacologic contraceptives including oral, parenteral and transcutaneous delivery; condoms with spermicide; diaphragm with spermicide; surgical sterilization; intrauterine device; abstinence; and post-menopause.
3. Currently breast-feeding (if female).
4. Behavioral, cognitive, or psychiatric disease that in the opinion of the investigator affects the ability of the participant to understand and cooperate with the study protocol.
5. Laboratory evidence of liver disease (alanine aminotransferase [ALT] greater than the upper limit of normal of the testing laboratory).
6. Laboratory evidence of renal disease (serum creatinine greater than the upper limit of normal of the testing laboratory).
7. Laboratory evidence of hematologic disease (absolute neutrophil count <1,500/mm^3; hemoglobin less than the lower limit of normal of the testing laboratory, by sex; or platelet count <140,000/mm^3).
8. Evidence of clinically significant neurologic, cardiac, pulmonary, hepatic, endocrine, rheumatologic, autoimmune or renal disease by history, physical examination, and/or laboratory studies including urinalysis (greater than trace protein or any glucose on urine dip will be confirmed negative prior to enrollment).
9. Other condition that in the opinion of the investigator would jeopardize the safety or rights of a participant participating in the trial or would render the subject unable to comply with the protocol.
10. Participation in another investigational vaccine or drug trial within 30 days of starting this study, or while this study is ongoing.
11. Participant has had medical, occupational or family problems as a result of alcohol or illicit drug use during the past 12 months.
12. History of a severe allergic reaction or anaphylaxis.
13. Positive ELISA and confirmatory Western blot tests for HIV-1.
14. Positive ELISA and confirmatory immunoblot tests for HCV.
15. Positive HBsAg by ELISA.
16. Known immunodeficiency syndrome.
17. Use of corticosteroids (excluding topical or nasal) or immunosuppressive drugs within 30 days of starting this study or while the study is ongoing.
18. Receipt of a live vaccine within past 4 weeks or a killed vaccine within past 2 weeks prior to entry into the study.

19. History of a surgical splenectomy.
20. Receipt of blood products within the past 6 months.
21. Previous receipt of an investigational malaria vaccine.
22. Receipt of antimalarial prophylaxis during the past 12 months.
23. Prior malaria infection.
24. Any medical, psychiatric, social, or occupational condition, or other responsibility that, in the judgment of the PI, would interfere with the evaluation of study objectives.
25. History of a known allergy to nickel.
26. Pre-existing autoimmune or antibody mediated diseases including but not limited to: systemic lupus erythematosis, rheumatoid arthritis, multiple sclerosis, Sjogren's syndrome, autoimmune thrombocytopenia; or laboratory evidence of possible autoimmune disease determined by a positive anti-dsDNA titer, positive rheumatoid factor, proteinuria (greater than trace protein), and/or a positive ANA titer of >1:80 at screening.
27. Receipt of chloroquine or other aminoquinolines within 12 weeks of study entry.

4.3 Treatments that could potentially interfere with vaccine-induced immunity:

The following criteria should be checked at each visit. If any become applicable during the study, the participant may be excluded from receiving further doses of the study vaccine. The participant will, however, be encouraged to remain in the safety evaluation for doses already received.

1. Use of any investigational drug or vaccine other than the study vaccine during the study period.
2. Administration of chronic (defined as more than 14 days) immunosuppressants or other immune-modifying drugs within six months of vaccination. (For corticosteroids, this will mean prednisone, or equivalent, greater than or equal to 10 mg/day. Topical steroids are allowed.)
3. Administration of a licensed vaccine during the period starting from day –14 to day 70 (14 days before and after each vaccination).
4. Administration of immunoglobulins and/or any blood products up to 30 days after the last dose of vaccine.

4.4 Contraindications to vaccination:

The following criteria should be checked prior to each immunization and are contraindications to further immunization. However, the participant will be encouraged to remain in the safety evaluation for doses already received.

1. Hypersensitivity reaction following administration of the study vaccine.
2. Positive urine β-hCG.
3. If a participant develops a positive anti-dsDNA test during the course of the trial, s/he will be ineligible to receive further vaccinations.

4.5 Indications for deferral of vaccination:

The following adverse events constitute grounds for deferral of vaccine administration at that point in time; if any one of these adverse events occurs at the time scheduled for vaccination, the participant may be vaccinated at a later date, within the allowable time interval specified in the protocol, or withdrawn at the discretion of the investigator. The participant must be followed

until resolution of the event as with any adverse event. If the participant is withdrawn from the study, he/she will be encouraged to remain in the safety evaluation for the duration of the study.

1. Oral temperature > 37.5°C at the time of vaccination will warrant deferral of immunization until fever and symptoms resolve.
2. Any other condition that in the opinion of the investigator poses a threat to the individual if immunized or that may complicate interpretation of the safety of the vaccine following immunization.

Such individual(s) will be followed daily in the clinic until the symptoms resolve or the window for immunization expires. No further vaccination will be performed if the participant does not recover (oral temperature $\leq$ 37.5°C and/or lack of symptoms) within 7 days of the originally scheduled vaccination date. The participant, however, will be followed for safety and immunogenicity. If the individual meets any of the above criteria for deferral on the day of first immunization the investigator may elect to exclude the participant from further participation in the study.

4.6 Participant withdrawal criteria:

A participant will not be considered to have completed the trial if any of the following reasons apply. However, any participant who has received at least one dose of vaccine will be encouraged to remain in the safety evaluation for the duration of the study.

1. Developed an adverse event - applies to a participant who is withdrawn from the study due to an adverse event, serious or otherwise.
2. Lost to follow-up - applies to a participant who consistently does not return for protocol study visits, is not reachable by telephone or other means of communication and/or is not able to be located.
3. Research terminated by sponsor or investigator - applies to the situation where the entire study is terminated by the sponsor, or investigator for any reason.
4. Withdrawal of Consent - applies to a subject who withdraws consent to participate in the study for any reason.
5. Non-compliant with protocol - applies to a participant who does not comply with protocol specific visits or evaluations, even though the participant is able to comply, on a consistent basis, such that adequate follow-up is not possible or the participant's safety would be compromised by continuing in the trial.
6. If participant becomes pregnant during the course of the trial.
7. If new safety data becomes available during the trial that impact participant health.
8. Other - is a category used when previous categories do not apply and requires an explanation.

5.0 VACCINE PREPARATION

5.1 Supplies

Research products for this protocol will be supplied to the study-site pharmacist by the Pharmaceutical Development Section, Pharmacy Department, Clinical Center, National Institutes of Health, where the vaccine was formulated and vialed. Temperatures during vaccine transport will remain between 0.5 to 9°C. The site pharmacist will label the kit with the

assigned Participant ID number. The vaccines and CPG should be stored in the refrigerator at 2 to 8°C and should not be frozen. Vials should be stored in the upright position. CPG 7909 will be supplied to the study site pharmacist by the Malaria Vaccine Development Branch, who has obtained the adjuvant through a clinical trials agreement with Coley Pharmaceuticals Group, Inc.

5.1.1 $MSP1_{42}$-C1/Alhydrogel®

$MSP1_{42}$-C1/Alhydrogel® malaria vaccine is supplied as a cloudy suspension in single-dose vials. Each 2.0 mL vial contains 0.7 mL, of which 0.5 mL is the intended volume to be injected. 0.5 mL of vaccine contains the equivalent of 404 µg of aluminum as Alhydrogel® (800 µg of aluminum hydroxide gel per dose) onto which either 40 µg or 160 µg of recombinant $MSP1_{42}$-C1 has been bound. The product conforms to established requirements for sterility, safety and identity.

5.1.2 CPG 7909

CPG 7909 (supplied by Coley Pharmaceuticals) is supplied as a solution in single use vials. The product conforms to established requirements for sterility, safety and identity. CPG 7909 is supplied as a 1.0 mL solution in 2.0 mL single use vial containing 10 mg/mL without preservative. A volume of 0.08 mL of CPG 7909 (10 mg/mL) will be withdrawn from the vial of CPG 7909 using a 300 µL insulin syringe. The 0.08 mL of CPG 7909 will be added to a vial containing 0.7 mL of $MSP1_{42}$-C1/Alhydrogel®. This point of injection formulation should yield a final injection volume of 0.55 mL to be withdrawn for each vaccination. The actual dose of injected CPG 7909 will therefore be 0.56 mg.

The mixture must be administered not more than 6 hours after the addition of CPG 7909. The point of injection formulation will be prepared by the trial site pharmacist. The syringe containing formulated vaccine will be labeled by the pharmacist with the participant's study ID and initials.

5.2 Vaccine Storage

$MSP1_{42}$-C1/Alhydrogel® vaccine, CPG 7909 and the formulated mixture should be maintained at 2 to 8°C until just prior to administration. Vaccine should NOT be frozen at any time.

5.3 Vaccine Accountability

Site pharmacists are responsible for maintaining an accurate inventory and accountability record of vaccine supplies for this study.

5.4 Disposition of Used/Unused Supplies

After administration of a vaccine dose, the single-dose vial will be returned to the Pharmacy at the test site, and vials will be accounted for and stored until monitoring by the IND sponsor. The empty vials of both $MSP1_{42}$-C1/Alhydrogel® vaccine and CPG 7909 may then be disposed of according to site protocol. At the conclusion of vaccine administration, all unused vaccine supplies will be returned to the MVDB.

6.0 STUDY PROCEDURES

The following sections provide a detailed listing of the procedures and blood draws. A total of 626.5 mL will be drawn over the duration of the trial.

6.1 Screening (up to 60 days prior to vaccination):

1. Explain the study and Informed Consent to the participant.
2. Ensure the participant has signed the Informed Consent and receives a signed copy of the Informed Consent and has passed the informed consent comprehension exam.
3. Explain the HIV Informed Consent and obtain a signed copy from the participant.
4. Elicit a complete medical history, including menstrual and contraceptive history and/or history of surgical sterility for females.
5. Administer a complete physical examination.
6. Obtain blood for hematology, biochemistry, anti-dsDNA, ANA, rheumatoid factor, and serologic tests for viral hepatitis and HIV in all participants.
7. Obtain urine for urine dipstick testing, as well as β-hCG testing in females.
8. Counsel females to avoid becoming pregnant during the study.

6.2 Randomization Process

The first 30 eligible participants will be randomly assigned to either Group A or Group B. Treatment assignment will be done by random number generator. The study numbers for each participant will be assigned in the order in which the participants are enrolled on the vaccination day. The randomization code will be prepared in advance of the start of the study and will contain sequential codes linking a study number to a vaccine assignment.

A master log of treatment assignment will be maintained in a record separate from other study records. This log will be kept by the study pharmacist. It will be kept in a locked room with limited access. The participants will be informed of their treatment assignment on the last study visit. Access to the randomization list will be exclusively limited to the pharmacist and his staff. On days of vaccination, only the pharmacist (and his staff) will have access to the vaccine preparation room. Between vaccination days, the randomization list will be stored in a locked cabinet in the vaccination preparation room. The pharmacist will not be blinded and will not be involved in evaluation of the participants. The medical monitor and DSMB will also keep one set of the randomization code in a sealed envelope in the event that emergency unblinding is required.

6.3 Screening Process and Enrollment

Volunteers will be recruited from the Baltimore/Washington area using a variety of sources including: volunteers previously enrolled in vaccine trials at the CIR; the use of a general screening protocol that has been in use at the CIR for more than 20 years and by the use of study-specific IRB-approved print and/or other media advertising.. Once the volunteer has been identified as being interested/appropriate for this study, the volunteer will undergo a study-specific screening visit during which the participant will read the consent form, be encouraged to ask questions, and then take and pass a true-false questionnaire to test consent comprehension. The participant must pass the questionnaire prior to being eligible for enrollment. Study staff will review incorrect answers with participants to ensure full comprehension of the study. The participant may either sign the consent form during the

screening visit, or return after further consideration. A participant will not be considered enrolled until they have received their first vaccination.

6.4 Immunization procedure:

Participants will receive three immunizations, on Days 0, 28, and 56. 0.50 mL of $MSP1_{42}$-C1/Alhydrogel® or 0.55 mL of $MSP1_{42}$-C1/Alhdyrogel® + CPG 7909 will be delivered by intramuscular injection (IM) in the deltoid muscle with a 22-gauge needle of appropriate length after preparation of the site with alcohol. Successive vaccinations will be given in alternating arms.

6.5 Clinical monitoring and evaluation:

See **Appendix A** for a tabular representation of study procedures. In addition, photographs may be taken of injection site reactions or rashes that may occur. The volunteer's face will not be included in the photograph nor will any identifying scars, tattoos, or other lesions that may identify the volunteer.

Study Day 0 (Day of First Vaccination)

1. Verify that Informed Consent was obtained.
2. Verify that all applicable eligibility criteria have been met.
3. Perform directed history and clinical assessment.
4. Obtain approximately 92 mL of blood for hematology, biochemistry, rheumatoid factor, anti-dsDNA, ANA, C3, C4, CH50, anti-$MSP1_{42}$ antibody ELISA, GIA and B/T cell analysis.
5. Obtain urine for urine dipstick testing.
6. For females, obtain a urine sample for β-hCG testing. Ensure that test is negative before proceeding; a positive test will exclude the participant from the trial.
7. Record vital signs (blood pressure, temperature, heart rate and respiratory rate).
8. Administer the vaccine.
9. Observe for at least 60 minutes after vaccination to evaluate for immediate adverse reactions.
10. Education by study staff during 60 minute post-immunization wait period describing proper use of digital thermometers, injection site reaction measurement tools and participant symptom diaries. Study staff will also discuss signs and symptoms of potential adverse events.

Study Day 1

1. Perform directed history and clinical assessment.
2. Record vital signs.

Study Day 3

1. Perform directed history and clinical assessment.
2. Record vital signs.
3. Obtain approximately 9 mL of blood for hematology and biochemistry tests.

Study Day 7 +/- 1

1. Perform directed history and clinical assessment.
2. Record vital signs.
3. Obtain approximately 52 mL of blood for hematology, anti-dsDNA, and B/T cell analysis. Aliquots of serum will be reserved for additional testing of ANA, rheumatoid factor, and anti-ssDNA if indicated (participant develops a positive anti-dsDNA).
4. Collect Day 0-6 diary card.

Study Day 14 +/- 2

1. Perform directed history and clinical assessment.
2. Record vital signs.
3. Obtain approximately 19 mL of blood for hematology, biochemistry, and anti-$MSP1_{42}$ antibody ELISA.
4. Obtain urine for urine dipstick testing

Study Day 28 +/- 7 (Day of Second Vaccination)

1. Perform directed history and clinical assessment.
2. Obtain approximately 46 mL of blood for hematology, biochemistry, anti-dsDNA, anti-$MSP1_{42}$ antibody ELISA and B/T cell analysis. Aliquots of serum will be reserved for additional testing of ANA, rheumatoid factor, and anti-ssDNA if indicated (participant develops a positive anti-dsDNA).
3. Obtain urine for urine dipstick testing
4. For females, obtain a urine sample for β-hCG testing. Ensure that test is negative before proceeding; a positive test will exclude the participant from the trial.
5. Record vital signs (blood pressure, temperature, heart rate, and respiratory rate).
6. Administer the vaccine.
7. Observe for at least 60 minutes after vaccination to evaluate for immediate adverse reactions.
8. Education by study staff during 60 minute post-immunization wait period describing proper use of digital thermometers, injection site reaction measurement tools and participant symptom diaries. Study staff will also discuss signs and symptoms of potential adverse events.

Study Day 29 (1 days after Second Vaccination)

1. Perform directed history and clinical assessment.
2. Record vital signs.

Study Day 31 (3 days after Second Vaccination)

1. Perform directed history and clinical assessment.
2. Record vital signs.
3. Obtain approximately 29 mL of blood for hematology and biochemistry tests, and B/T cell analysis.

Study Day 35 +/- 1 (7 days after Second Vaccination)

1. Perform directed history and clinical assessment.
2. Record vital signs.

3. Obtain approximately 36 mL of blood for hematology, anti-dsDNA, and B/T cell analysis. Aliquots of serum will be reserved for additional testing of ANA, rheumatoid factor, and anti-ssDNA if indicated (participant develops a positive anti-dsDNA).
4. Collect Day 0-6 diary card.

Study Day 42 +/- 2 (14 days after Second Vaccination)

1. Perform directed history and clinical assessment.
2. Record vital signs.
3. Obtain approximately 39 mL of blood for hematology, biochemistry, anti-$MSP1_{42}$ antibody ELISA, and GIA.
4. Obtain urine for urine dipstick testing

Study Day 56 +/- 7 (Day of Third Vaccination)

1. Perform directed history and clinical assessment.
2. Obtain approximately 66 mL of blood for hematology, biochemistry, anti-dsDNA, anti-$MSP1_{42}$ antibody ELISA, GIA and B/T cell analysis. Aliquots of serum will be reserved for additional testing of ANA, rheumatoid factor, and anti-ssDNA if indicated (participant develops a positive anti-dsDNA).
3. Obtain urine for urine dipstick testing.
4. For females, obtain a urine sample for β-hCG testing. Ensure that test is negative before proceeding; a positive test will exclude the participant from the trial.
5. Record vital signs (blood pressure, temperature, heart rate, and respiratory rate).
6. Administer the vaccine.
7. Observe for at least 60 minutes after vaccination to evaluate for immediate adverse reactions.
8. Education by study staff during 60 minute post-immunization wait period describing proper use of digital thermometers, injection site reaction measurement tools and participant diaries. Study staff will also discuss signs and symptoms of potential adverse events.

Study Day 57 (1 days after Third Vaccination)

1. Perform directed history and clinical assessment.
2. Record vital signs.

Study Day 59 (3 days after Third Vaccination)

1. Perform directed history and clinical assessment.
2. Record vital signs.
3. Obtain approximately 29 mL of blood for hematology and biochemistry tests and B/T cell analysis.

Study Day 63 +/- 1 (7 days after Third Vaccination)

1. Perform directed history and clinical assessment.
2. Record vital signs.
3. Obtain approximately 32 mL of blood for hematology, anti-dsDNA, and B/T cell analysis. Aliquots of serum will be reserved for additional testing of ANA, rheumatoid factor, and anti-ssDNA if indicated (participant develops a positive anti-dsDNA).

4. Collect Day 0-6 diary card.

Study Day 70 +/- 2 (14 days after Third Vaccination)

1. Perform directed history and clinical assessment.
2. Record vital signs.
3. Obtain approximately 39 mL of blood for hematology, biochemistry, anti-MSP1_{42} antibody ELISA, and GIA.
4. On selected volunteers, plasmapheresis will be arranged at the National Institutes of Health for preparation of serum standards
5. Obtain urine for urine dipstick testing.

Study Day 84 +/- 14 (28 days after Third Vaccination)

1. Perform directed history and clinical assessment.
2. Obtain approximately 22 mL of blood for hematology, anti-dsDNA, and anti-MSP1_{42} antibody ELISA. Aliquots of serum will be reserved for additional testing of ANA, rheumatoid factor, and anti-ssDNA if indicated (participant develops a positive anti-dsDNA).
3. Obtain urine for urine dipstick testing.

Study Day 140 +/- 14 (84 days after Third Vaccination)

1. Perform directed history and clinical assessment.
2. Obtain approximately 34 mL of blood for hematology, B/T cell analysis and anti-MSP1_{42} antibody ELISA.

Study Day 236 +/- 30 (180 days after Third Vaccination)

1. Perform directed history and clinical assessment.
2. Record vital signs.
3. Obtain approximately 62 mL of blood for hematology, anti-dsDNA, GIA and anti-MSP1_{42} antibody ELISA and B/T cell analysis.
4. Obtain urine for urine dipstick testing. Aliquots of serum will be reserved for additional testing of ANA, rheumatoid factor, and anti-ssDNA if indicated (participant develops a positive anti-dsDNA).

Participants who are found to have a high serum antibody response to MSP1_{42} by ELISA at study day 42 (14 days after the second vaccination) may be invited to undergo plasmapheresis around the scheduled study day 70 blood draw. These participants will undergo an additional informed consent procedure and will sign an additional informed consent form for plasmapheresis. Continued participation in the study described in this protocol will NOT be dependent on their agreement to undergo plasmapheresis. The necessity of this additional amount of blood will be determined upon reviewing the ELISA values from day 42. These extra serum samples would be used to create a high titer reference standard reagent to be used in future MSP1_{42} vaccine trials. Serum samples of persons vaccinated with MSP1_{42} would be compared against this reference standard for better comparison of vaccine immunogenicity between trials. The testing of vaccine efficacy in this and future trials of MSP1_{42} will rely on the preparation of a well-defined reference standard reagent against which the sera from vaccinate participants can be tested and compared. An accepted approach for making such a reference reagent is to make a pool of sera with known anti-MSP1_{42}-C1 antibody levels which is then used

as a reference serum in ELISA assays. Such a reference serum would ideally be prepared in a large enough volume to be stored and used for several years. This reference serum will be used for research purposes only and will not have any commercial use or value.

6.6 Participant symptom diary:

Participants will be asked to keep daily symptom diaries recording oral temperature once during the day, as well as pain, tenderness, redness, and swelling at the injection site for 6 days following each immunization. The size of any injection site reaction will be measured utilizing a standardized clear plastic measurement device and recorded in the participant symptom diary.

6.7 Laboratory testing:

Using standard techniques, the clinical laboratory, will perform the following tests:

1. Complete blood count plus white blood cell differential*
2. Serum creatinine
3. Alanine aminotransferase (ALT)
4. HIV assay (FDA-approved screening antibody assay with Western Blot confirmation)
5. HBsAg ELISA
6. HCV assay (FDA-approved screening antibody assay and immunoblot confirmation or viral PCR confirmation)
7. Urinalysis (in the event of an abnormal urine dipstick test)
8. Rheumatoid factor
9. Anti-dsDNA (ELISA)
10. ANA
11. C3
12. C4
13. CH50

*The following CBC parameters will be assessed for safety throughout the trial: WBC, ANC (absolute neutrophil count), Hemoglobin and Platelet count. Should a volunteer develop a clinically significant hematologic abnormality such as anemia, leucopenia, or leukocytosis, additional laboratory assays will be done to assess the etiology of the abnormality. These may include examination of peripheral blood smear, iron binding capacity, serum iron level, ferritin, direct Coombs test, cultures, other markers of inflammation.

Urine β-hCG testing will be performed at the clinical trial site, using an FDA-approved urine pregnancy test kit. Urine dipstick testing will be performed at the clinical trial site using an FDA-approved product.

Anti-$MSP1_{42}$ ELISAs and GIAs will be performed at the MVDB in Rockville, Maryland. Additionally, the presence and quantity of antigen-specific B cells and CD4 and CD8 T cells producing specified cytokines following vaccination will be performed at the MVDB.

Anti-ssDNA will be performed by the Coley Pharmaceutical Group. This test is a research assay that will be performed in a GLP lab and will be performed only on serum from participants who develop a positive anti-dsDNA test during the course of the trial.

6.8 Immunologic testing:

6.8.1 Antibody assay (ELISA):

Antibody levels to the $MSP1_{42}$ antigen will be measured in serum by ELISA. Briefly, microwell plates (Dynex Technologies) are coated overnight at 4°C with 100 µl/well of antigen solution (1 µg/ml). Plates are washed with TRIS-buffered saline (TBS) containing 0.1% Tween-20 (0.1% T-TBS) and blocked with TBS containing 5% skim milk powder for two hours at room temperature. After washing with 0.1% T-TBS, serum samples in TBS containing 5% skim milk are added in triplicate and incubated with $MSP1_{42}$-coated plates for 2 hours at room temperature. After incubation, unbound antibodies are removed by washing the plates with 0.1% T-TBS, and 100 µl of alkaline phosphatase-conjugated goat anti-human IgG solution (Kirkegaard & Perry Labs, Gaithersburg, MD, 1:1000 dilution in 0.5% T-TBS containing 1% BSA,) is added to each well and incubated for 2 hours at room temperature. Plates are then washed with 0.1% T-TBS, followed by adding 100 µL of substrate solution (Sigma 104 substrate, St. Louis, MO) to each well; the plates are then covered with aluminum foil and incubated for 20 minutes at room temperature for color development. The plates are read immediately at 405 nm with a microplate reader (Spectramax 340PC Molecular Devices). A serially diluted standard serum is run on each plate and will initially be obtained from a pool of human serum from malaria-endemic area of Mali standardized for anti-$MSP1_{42}$ IgG. This serum is assigned a unit value as the reciprocal of the dilution giving an O.D. = 1 on an $MSP1_{42}$-coated plate. Using the standard curve, the absorbance of individual test sera is converted to antibody units (SOFTmax PRO ver. 3; Molecular Devices Co.). Similar ELISA analysis will also be performed for vaccine induced antibodies reactivity to $MSP1_{19}$ and $MSP1_{33}$.

6.8.2 In vitro Parasite Growth inhibition assay (GIA):

Late trophozoite and schizont stages of *P. falciparum* (FVO and 3D7 lines) parasitized erythrocytes are collected by Percoll gradient and/or 5% sorbitol treatment. The synchronized parasites are diluted with human RBCs to give a final concentration of 0.3% parasitemia, 1% hematocrit in growth medium (RPMI 1640 containing 10% human O+ serum, 25 mM HEPES, 0.4 mM hypoxanthine, 30 mM sodium bicarbonate and 25 mg/L of gentamicin). Preliminary tests may be performed with test serum which has been heat inactivated (at 56°C for 20 minutes) and pre-adsorbed with uninfected human O^+ RBCs to remove anti-human immunoglobins. However, definitive studies are performed with IgG purified from human serum. Serial dilutions of immune serum or IgG are mixed with parasitized erythrocytes in 96-well tissue culture plates. Controls include autologous pre-immune serum (20%), human AB^+ serum (positive control), and uninfected erythrocytes (negative control). *P. falciparum* cultures are grown in 5% O_2, 5% CO_2, and 90% N_2 at 37°C for 40hrs. After mixing, 50µl samples are transferred to C-bottom 96-well plates containing 250 µl of cold PBS. Plates are then centrifuged to pellet the cells and frozen for at least 3 hours to lyse the cells. Relative parasitemia levels are determined by means of a colorimetric measurement of parasite lactate dehydrogenase (pLDH) activity. Test samples are dissolved in pLDH substrate buffer (100 uL of 100 mM Tris, pH 8.0, containing 50 mM sodium L-lactate, 0.25% TritonX-100, 0.075 mM 3-acetylpyridine adenine dinucleotide(APAD), 1 U/ml diaphorase, 20 µg Nitro Blue Tetrazolium (all reagents from Sigma Chemical, St. Louis, MO)) and color allowed to develop in the dark for 30 minutes at room temperature. Absorbance at 650 nm is then determined using a Spectra Max 340PC (Molecular Devices) plate reader. Percent growth inhibition is calculated by the formula: 100% - [(A_{650} immune sample - A_{650} normal RBC only) / (A_{650} Pre-immune control - ABS_{650} RBC only) X 100%]. All assays are run in triplicate.

6.8.3 Assessment of cellular immune responses

Blood will be obtained from participants prior to vaccination and at specified time points after vaccination for assessment of B- and T-cell responses to $MSP1_{42}$ (See Appendix A). These samples will be assayed for evidence of B Cell immunologic memory specific for $MSP1_{42}$ antigens. Additionally, malaria-specific T cell effector cytokines will be evaluated by three single-color cytokine (IL2, IL4, IFN-γ) ELISPOT assays of PBL. Finally, production of multiple cytokines and chemokines by antigen-specific T cells will also be assessed by in vitro stimulation of PBL with $MSP1_{42}$-FVO and $MSP1_{42}$-3D7 proteins using a quantitative multiplex bead ELISA (Luminex) system.

6.9 Use, Storage, and Tracking of Specimens and Data

Samples and data collected under this protocol will be used to study malaria and related diseases, and possible adverse reactions to vaccination. No genetic testing will be performed. Access to research samples will be limited using either a locked room or a locked freezer. Samples and data will be stored using codes assigned by the investigators or their designees. Data will be kept in password-protected computers. Only investigators or their designees will have access to the samples and data.

Samples will be stored at the MVDB in Rockville, MD or at MVDB's designated repository, Thermo Scientific, Rockville, MD. Samples will be tracked using a software database, e.g. Freezerworks. Any loss or unanticipated destruction of samples (for example, due to freezer malfunction) or data (for example, misplacing a printout of data with identifiers,) will be reported to the IRBs. Such a loss will be reported to the NIAID IRB as a protocol violation under the following classification: The violation compromises the scientific integrity of the data collected for the study.

6.10 Retention of Specimens for Future Use

All specimens collected as part of this trial may, with the subject's permission, be stored for future research. Whether or not a volunteer agrees to storage of specimens will not affect his/her ability to participate in this trial. These samples may be used to learn more about malaria infection and other diseases. These samples will not be sold or used to make commercial products. Samples will only be stored with the volunteer's permission. The volunteer may withdraw permission for future use of specimens at any time. If a volunteer withdraws his or her permission for future use of specimens, those specimens will be destroyed. All samples stored will be labeled with the volunteer's study identification (ID) number, which cannot identify the study subject but is linkable to other research databases (e.g., from questionnaires, clinical assessments, logbooks, etc.) generated by the main study. The database will contain only the study volunteer's ID number. A master log linking the study volunteer ID number to the name of the volunteer will be maintained in a password protected database system with access limited to authorized research team members.

At the completion of the protocol (termination), samples and data will either be destroyed, or after IRB approval, transferred to another existing protocol or a repository. In the future, other investigators (both at NIH and outside) may wish to study these samples and/or data. In that case, IRB approval must be sought prior to any sharing of samples. Any clinical information shared about the sample with or without patient identifiers would similarly require prior IRB

approval. The research use of stored, unlinked or unidentified samples (for example, as a standard for immunological analyses), may be exempt from the need for prospective IRB review and approval. Exemption requests will be submitted in writing to the NIH Office of Human Subjects Research, which is authorized to determine whether a research activity is exempt.

7.0 ADVERSE EVENTS MONITORING AND REPORTING

7.1 Definitions:

7.1.1 Adverse Event (AE):

Any untoward medical occurrence in a trial participant administered the experimental vaccine and that does not necessarily have a causal relationship with this vaccination. An AE can therefore be any unfavorable and unintended sign (including an abnormal laboratory finding), symptom, or disease temporally associated with the use of the investigational vaccine, whether or not related to it. This includes an exacerbation of pre-existing conditions and concurrent illnesses. All adverse events must be graded for intensity and relationship to the investigational vaccine as described in Sections 7.2.2 and 7.2.3. The occurrence of an adverse event may come to the attention of study personnel during study visits and interviews or by a vaccine recipient presenting for medical care.

7.1.2 Serious Adverse Event (SAE):

An adverse event, whether considered related to the investigational vaccine or not, meeting one of the following conditions:

1. Death during the period of protocol-defined surveillance
2. Life threatening: defined as an event that places a subject at immediate risk of death at the time of the event and does not refer to an event that hypothetically might have caused death were it more severe
3. Hospitalization during the period of protocol defined surveillance: defined as at least an overnight stay in the hospital or emergency ward for treatment that would have been inappropriate if administered in the outpatient setting
4. Results in a congenital anomaly or birth defect
5. Results in a persistent or significant disability or incapacity: defined as a substantial disruption of the study participant's ability to carry out normal life functions
6. Any other important medical event that may not result in death, be life threatening, or require hospitalization, may be considered a serious adverse event when, based upon appropriate medical judgment, the event may jeopardize the subject and may require medical or surgical intervention to prevent one of the outcomes listed above

7.2 Assessment of adverse events:

7.2.1 Identification of adverse events:

Assessment of safety will include clinical observation and monitoring of hematological, chemical and immunologic parameters. Safety will be evaluated by monitoring of participants for local and systemic adverse reactions during the course of the trial. Participants will be closely monitored for at least 60 minutes following each immunization. Additionally, participants will

return to the clinic on Days 1, 3, 7 and 14 following each vaccination for clinical assessments. Participants will be asked to keep daily symptom diaries for 6 days after each immunization, recording oral temperatures once daily, as well as a subjective assessment of the extent of swelling, erythema, and pain at the site of injection. The size of any injection site reaction will be measured utilizing a standardized clear plastic measurement device and recorded in the participant symptom diary. All adverse events will be graded for intensity and relationship to study product. Reactions will be graded as described in Section 7.2.2. A study clinician will be available by telephone or pager 24 hours a day during the study evaluation period.

The clinical significance of laboratory test abnormalities will be assessed and will not be recorded as an adverse event unless the abnormality is considered clinically significant.

7.2.2 Determination of severity:

All AEs will be assessed by a study investigator using the following protocol-defined grading system:

Grade 0 (none)	
Grade 1 (mild):	No effect on activities of daily living
Grade 2 (moderate):	Partial limitation in activities of daily living (can complete $\geq$ 50% of baseline), or treatment given
Grade 3 (severe):	Activities of daily living limited to < 50% of baseline

Intensity of the following adverse events will be assessed by the investigator as described in **Table 6-2**. All safety laboratory AEs will be graded in severity following the toxicity table in Appendix B. Unexpected adverse events and clinically significant laboratory abnormalities not described in Table 6-2 or Appendix B will be graded according to Appendix C.

Table 6-2: Assessment of Adverse Event Intensity

Adverse event	Intensity	Intensity
Pain at injection site	0	Absent
	1	Pain which is easily tolerated
	2	Pain that interferes with daily activity
	3	Pain that prevents daily activity
Erythema at injection site	0	0 mm
	1	>0 - ≤20 mm
	2	>20 - ≤50 mm
	3	>50 mm
Swelling at injection site	0	0 mm
	1	>0 - ≤20 mm
	2	>20 - ≤50 mm
	3	>50 mm
Induration at injection site (as determined by clinical assessment)	0	0 mm
	1	>0 - ≤20 mm
	2	>20 - ≤50 mm
	3	>50 mm
Fever (oral)	0	≤99.5°F (≤37.5°C)
	1	>99.5 - 100.4°F (>37.5 – 38°C)
	2	>100.4 - 102.2°F (>38 – 39°C)
	3	>102.2°F (>39°C)
Headache	0	None
	1	Headache which is easily tolerated
	2	Headache that interferes with daily activity
	3	Headache that prevents daily activity
Nausea	0	None
	1	Nausea that is easily tolerated
	2	Nausea that interferes with daily activity
	3	Nausea that prevents daily activity
Malaise	0	None
	1	Malaise which is easily tolerated
	2	Malaise that interferes with daily activity
	3	Malaise that prevents daily activity
Myalgia	0	None
	1	Myalgia which is easily tolerated
	2	Myalgia that interferes with daily activity
	3	Myalgia that prevents daily activity
Arthralgia	0	None
	1	Joint pain which is easily tolerated
	2	Joint pain that interferes with daily activity
	3	Joint pain that prevents daily activity
Urticaria	0	None
	1	Requiring no medications
	2	Requiring PO or topical treatment or IV medication or steroids for < 24 hours
	3	Requiring IV medication or steroids for >24 hours

7.2.3 Association with receipt of the study vaccine:

All adverse events will have their possible relationship to study vaccine assessed using the following terms:

Definitely: clear-cut temporal association, and no other possible cause.
Probably: clear-cut temporal association and a potential alternative etiology is not apparent.
Possibly: less clear temporal association; other etiologies also possible.
Unlikely: temporal association between the AE and the vaccine or the nature of the event is such that the vaccine is not likely to have had any reasonable association with the observed illness/event (cause and effect relationship improbable but not impossible).
Unrelated: the AE is completely independent of vaccine administration; and/or evidence exists that the event is definitely related to another etiology.The degree of certainty with which an adverse event can be attributed to administration of the study vaccine will be determined by how well the event can be understood in terms of one or more of the following:

1. The event being temporally related with vaccination or reproduced on re-vaccination.
2. A reaction of similar nature having previously been observed with this type of vaccine and/or formulation.
3. The event having often been reported in the literature for similar types of vaccines.

All local (injection site) reactions will be considered causally related to vaccination.

7.4 Adverse event reporting:

All SAEs will be reviewed by a study physician, recorded on the appropriate SAE form, and followed through to resolution by a study physician. All SAEs will be reported by telephone or fax within 1 working day of notification of the SAE occurrence to all of the following:

- IND Sponsor, Regulatory Compliance and Human Subjects Protection Branch Safety Section (RCHSPB Safety)/NIAID: Phone: 301-846-5301, Fax: 301-846-6224; email: RCHPSafety@mail.nih.gov
- Western IRB Phone: 800-562-4789, Fax: 360-252-2498
- NIAID IRB: Phone: 301-451-5147 or 301-451-5143, Fax: 301-480-6606

Additionally, any SAE that meets the stopping criteria or is otherwise defined as reportable to the DSMB in **Section 7.5.2** will be reported to the Executive Secretary of the DSMB (who is the same as the IND sponsor seen in this section). The Executive Secretary will notify the DSMB.

- NIAID Data, Safety and Monitoring Board (DSMB): Phone: 301-846-5301, Fax: 301-846-6224.

In addition, the NIAID investigators will provide copies of all SAEs occurring during this trial to the Safety Contact at Coley Pharmaceutical Group, Inc. The NIAID investigators shall forward to Coley (by fax, e-mail or overnight delivery) all SAE reports that NIAID provides to the FDA. These reports will include related as well as unrelated SAE reports. All SAE reports sent to Coley will include the investigator's assessment of causality. Reports will be sent to the Coley Safety Contact:

Mary Rice, Ph.D.
93 Worcester Street, Suite 101
Wellesley, MA 02481
Phone: 781-431-9000 x1251, Fax: 781-431-0941

E-mail: mrice@coleypharma.com

If the SAE report is telephoned to any of the above, a written SAE form will be submitted as soon as possible, but within 3 working days. The investigator will submit any follow up information missing in all SAE forms as soon as possible.
Following notification from the investigator, the Investigational New Drug (IND) sponsor, will report events that are both serious and unexpected that are possibly, probably or definitely related to the vaccine, to the FDA within the required timelines: fatal and life-threatening events within 7 calendar days (by phone or fax) and all other SAEs in writing within 15 calendar days. All SAEs not listed as possibly, probably, or definitely related will be reported to the FDA at least annually in a summary format.

In addition, the Principal Investigator will notify the MVDB within 1 working day of notification of the SAE occurrence.

All adverse events not judged to be serious will be reported to the IRBs and FDA at least annually in a summary format. All local and systemic reactions not meeting the criteria for "serious adverse events" will be captured on the appropriate case report form (CRF). These events will be followed to resolution.

7.5 Adverse event monitoring:

7.5.1 Medical monitor:

An independent medical monitor, Dr. Wellington Sun, MD, has been appointed for oversight of safety in this trial. The medical monitor will be available to advise the investigators on trial-related medical questions or problems, and act as a representative for the participants' welfare. Additionally, the medical monitor may ask to convene a safety meeting for review of any safety issue or adverse event.

7.5.2 Data Safety and Monitoring Board (DSMB):

Because this is a randomized and blinded study, NIAID policy mandates that it be reviewed by the permanent NIAID DSMB. This DSMB has been constituted to review the data and analysis plans of all intramural NIAID clinical studies that require DSMB oversight, and consists of experts in infectious diseases, biostatistics, and clinical trials. In addition, the medical monitor may be added as an ad hoc member for the duration of the study, at the discretion of the DSMB Chair, to review specific protocols if additional expertise is desired. The DSMB will serve in an advisory capacity to the investigators, the sponsor and the participating IRBs, which will consider its recommendations seriously in deciding whether or not the study may proceed. The Board meets at regular periods during the year, but has been empowered to convene in person or via teleconference between their regularly scheduled meetings should the need arise.

This protocol will be submitted to the DSMB for their review. All cumulative safety data reports from the trial will be submitted to the Board before dose escalation. The Safety data report will include data from all 3 vaccinations of the lower dose groups (up to and including study day 63) for the DSMB to review for dose escalation. Additional safety and immunology results and reports will be submitted to the DSMB as they become available. A final report will be submitted to the DSMB following study completion.

The DSMB will review cumulative safety data for evidence of study-related AEs, adherence to the protocol, and factors that may affect outcome or study data such as protocol violations and losses to follow up. Conference calls between the investigators and the DSMB will be scheduled within the week prior to dose escalation (i.e. after the third vaccination has been administered to Groups A & B, but before the first vaccination is administered to Groups C & D). If no stopping criteria are met (Section 7.6), dose escalation will proceed with approval from the DSMB. Should the DSMB not be able to meet, either in person or via teleconference, prior to scheduled dose escalation, the safety reports may be distributed to individual DSMB members who will then submit their vote for or against dose escalation; the DSMB Chair will tally the votes and communicate the collective decision to the Principal Investigator. If none of these options are possible, the DSMB may reserve the right to defer to the independent medical monitor to decide whether or not dose escalation may proceed. If a deferral to the medical monitor occurs, the IRBs will be promptly notified.

Written approval (via letter or email) to proceed to the next dose of vaccine must be obtained from the DSMB (or medical monitor in the event that the DSMB cannot review the safety data in a timely manner, as described above) prior to administration. Both the DSMB and medical monitor will have access to the randomization code, as they may wish to review the data in an unblinded fashion should significant safety questions arise prior to the final unblinding.

It is the Principal Investigator's (or designated agent) responsibility to ensure that the DSMB review the current safety data, study protocol, and any other requested documents at its meetings. Occurrence of an SAE that is both unexpected and possibly, probably, or definitely related to vaccination will be reported to the DSMB at the same time it is reported to the IRBs. Additionally, any new information that may adversely affect the safety of the participants or the conduct of the study will be submitted to the DSMB as it becomes available.

7.6 Stopping Criteria:

If a dose of vaccine is considered unacceptably reactogenic, dose escalation and/or additional vaccinations will be suspended until reviewed by the DSMB and study sponsor. This decision will also be reported to the NIAID and Western IRBs. The IRBs will be notified of the decision to halt or to continue vaccinations within one working day of the investigators becoming aware of such events.

The following criteria will be used to define unacceptable reactogenicity of the $MSP1_{42}$-C1/Alhydrogel® +/- CPG malaria vaccines:

1. One or more participants experience a SAE (as defined in **Section 7.1.2** in this protocol) that is determined to be possibly, probably or definitely related to the vaccine (as defined in **Section 7.2.3** in this protocol), **OR**
2. One or more participants experience a hypersensitivity reaction (a Grade 3 allergic reaction, as defined in Appendix C) that is possibly, probably or definitely related to the vaccine, **OR**
3. Any severe clinical illness occurs that is not explained by a diagnosis that is unrelated to vaccination, **OR**
4. One or more participants in a single-dose cohort experience an objective physical finding or safety laboratory abnormality of Grade 3 or higher (with the exception of injection site erythema, swelling, or induration), that is possibly, probably or definitely related to vaccine, **OR**

5. Two or more participants in a single dose cohort experience a Grade 3 clinical AE or a Grade 2 or higher safety laboratory abnormality, with the exception of Grade 2 neutropenia that completely resolves within 7 days, that is possibly, probably or definitely related to vaccine.

7.7 Breaking the study blind

This study will be double-blinded. The study will be unblinded to non-clinical investigators after participants in groups C and D reach study day 70. Unblinding at this time point will be done in order to evaluate the safety and immunogenicity of $MSP1_{42}$-C1/Alhydrogel® +/- CPG malaria vaccine in preparation for future vaccine trials. The randomization may be unblinded prior to this time for safety purposes only. This is very unlikely to occur, as once a vaccine is administered, knowing which vaccine was given is unlikely to influence the medical management of an adverse event. This procedure is therefore exceptional and any decision to unblind prior to study day 70 of groups C and D will be discussed with the sponsor, PI, scientific investigators, medical monitor and the DSMB. If deemed necessary for urgent safety reasons, the medical monitor, in consultation with the DSMB (if possible in a timely manner), may unblind a specific participant without revealing the study blind to the investigators and the sponsor. The medical monitor may put the study on hold at any time and discuss with the DSMB. The decision to completely unblind or permanently stop the study prior to the time-point stated in the protocol, Section 3.4, will take the final form of a formal recommendation by the DSMB to the study sponsor. The PI must then notify the IRBs of this decision.

In the event that the investigators come to know the study code prior to final unblinding, the PI must notify the sponsor immediately. The reasons will be documented by the PI and added to the study file.

Unblinding of the non-clinical investigators will be done after participants in groups C and D have reached study day 70..

8.0 DATA COLLECTION AND MONITORING

8.1 Source Documentation:

Complete source documentation (test results, procedure reports, hospital or medical records, etc.) is required for every study subject for the entire duration of the study. Case Report Forms (CRFs), participant symptom diaries and spreadsheets will be used to record data for subjects enrolled in the study. The Investigator is responsible for the accuracy and completeness of the data reported to the Sponsor in the CRFs, spreadsheets and diaries. Data reported in the CRFs and spreadsheets, derived from source documents, should be consistent with source documents or the discrepancies should be explained.

8.2 Study Documentation:

Study-related documentation will be completed as required by the Institutional Review Boards, the Sponsor and regulatory authorities. Annual reports to the IRBs will be submitted by the Investigator prior to the individual IRB deadlines. An annual report will also be submitted by the Sponsor to the FDA within 60 days of the anniversary date that the IND for $MSP1_{42}$-C1/Alhydrogel® +/- CPG malaria vaccine went into effect. These reports will provide a brief

description of the progress of the investigation as outlined in 21 CFR 312.33 and will include any revisions of the protocol.

The Investigator will maintain adequate records of the disposition of the investigational product, including dates, quantity and use by subjects. If the study is terminated, suspended or completed, the Investigator will return all unused supplies of the investigational product to the Sponsor.

8.3 Retention of Records:

Trial-related documents will be maintained by the Investigator for a period of two years after final marketing approval of the vaccine. If an application is not approved, the records and reports will be maintained until 2 years after shipment and delivery of the agent for investigational use is discontinued and FDA has been so notified. The Sponsor is required to inform the Investigator when such documents need no longer be retained. Storage of all trial-related documents will be such that confidentiality will be strictly maintained.

8.4 Protocol Revisions:

Any change to the protocol will be submitted to the participating IRBs (NIAID and trial site) and IND sponsor as a protocol amendment, and changes not affecting risk to participants may be expedited, as appropriate. All protocol amendments must be submitted to the participating IRBs and be approved before implementation unless there is a medical emergency. In the event of a medical emergency, the Investigator shall perform any medical procedures that are deemed medically appropriate. The Investigator must notify the IND Sponsor and participating IRBs (and FDA) of all such occurrences via a protocol amendment as soon as possible after the event.

Clinical Investigators' Brochure:

Investigators will receive the current version of the Clinical Investigators' Brochure, which comprehensively describes all the available preclinical and clinical experience with the experimental vaccine. If relevant new information becomes available during the course of the trial, the Investigators will receive a revised Investigators' Brochure or an amendment to the current version.

Study Monitoring:

The Sponsor will monitor all aspects of the study, with respect to current Good Clinical Practices, for compliance with applicable government regulations. Prior to the start of the study, the Investigator will be informed of the frequency of monitoring visits and will be given reasonable notification prior to each visit. The objectives of a monitoring visit will be to verify the prompt reporting of SAEs, to check the availability of the signed Informed Consent, and to compare CRFs and spreadsheets with source data for completeness and accuracy. During the monitoring visit, the Investigator (and/or designee) and other study personnel should be available to discuss the study. Study documents must be available for review throughout the course of the study. The Sponsor will retain originals of the FDA Form 1572 and copies of other study documents as deemed necessary.

9.0 STATISTICAL CONSIDERATIONS

9.1 Description of the statistical methods to be employed:

This study, like other Phase 1 studies, is basically exploratory rather than confirmatory; its purpose is to estimate event rates and patterns of immune responses rather than to test formal statistical hypotheses. Estimates will be presented with their 95% confidence intervals. Descriptive approaches will be used to meet the protocol objectives as stated in Section 2.0 of this protocol, as well as formal statistical tests as outlined below. Results will be presented in tabular format, as well as graphically where appropriate.

Primary Objective 1: To assess the safety, reactogenicity & immunogenicity of the $MSP1_{42}$-C1/Alhydrogel® +/- CPG vaccines at each dose

a. Summarize the frequency of systemic and local AEs.

b. Line listing of individual clinical and clinically significant laboratory AEs classified as systemic or local will be displayed in tabular format and stratified by dose cohort.

c. AEs will be summarized by severity and relationship to vaccine by individuals and dose cohort.

d. The proportion of participants with at least one local adverse event will be compared by dose cohort for each vaccine. Formal statistical tests will assess whether the 4 cohorts differ with respect to these proportions, and whether there is a dose-response relationship. To see if there is a difference in adverse events between the initial vaccination and the subsequent vaccinations we will perform a sign test, where the response for each participant is the difference between the number of adverse events following each vaccination.

e. To determine if CPG 7909 changes serum antibody levels to homologous antigen at day 70 (2 weeks after the third immunization).
    i. To compare the CPG 7909 groups to those without CPG 7909, we will use a stratified Wilcoxon test to check for significant differences in the antibody concentrations of the 2 dose groups. The test will be performed at the 0.05 level.

Secondary Objective 1: To demonstrate that the addition of CPG 7909 improves the specific immune responses to $MSP1_{42}$-FVO and $MSP1_{42}$-3D7, as compared to $MSP1_{42}$-C1/Alhydrogel® at day 70.

a. Mann-Whitney tests will be used to compare the antibody concentrations between groups. Specifically, the participants receiving 40 μg $MSP1_{42}$-C1/Alhydrogel® + CPG 7909 will be compared to those receiving only 40 μg $MSP1_{42}$-C1/Alhydrogel® and participants receiving 160 μg $MSP1_{42}$-C1/Alhydrogel® + CPG 7909 will be compared only to those receiving 160 μg $MSP1_{42}$-C1/Alhydrogel®.

Secondary Objective 2: To determine the dose of $MSP1_{42}$-C1/Alhydrogel® + CPG 7909 that generates the highest serum antibody levels to $MSP1_{42}$-FVO and $MSP1_{42}$-3D7 at day 70.

a. A Mann-Whitney test will be performed on the two dose levels.

Tertiary Objective 1: To measure the biological activity of the IgG purified from the antisera by an in vitro parasite growth inhibition assay using FVO and 3D7 parasites.

a. Growth inhibition, expressed as a percent of inhibition comparing test sera to pre-immune sera, will be displayed graphically.

Tertiary Objective 2: To determine the fine specificities and functionality of vaccine-induced antibody as judged by ELISA, GIA and IFA.

a. To determine the relationship between anti-$MSP1_{42}$ antibody concentration and degree of in vitro growth inhibition of *P. falciparum* in a GIA.
b. Rank correlation between ELISA values and growth inhibition will be assessed by the Spearman Rank correlation test.
c. Growth inhibition values will be plotted graphically as a function of ELISA units of antibody of each serum or IgG sample and stratified by dose.
d. To determine the relative specificity of the antibodies to a range of MSP1 serotypes, in addition to FVO and 3D7, as judged by ELISA and growth inhibition on a select panel of parasites with typed MSP1.
e. To determine the distribution of the specific $MSP1_{42}$ antibody response between the $MSP1_{33}$ and $MSP1_{19}$ regions.
f. To determine the ability of vaccine induced antibodies to recognize native parasite MSP1 protein by immunofluorescence assay (IFA).

Tertiary Objective 3: To assess and compare the duration of antibody responses to $MSP1_{42}$-FVO and $MSP1_{42}$-3D7.

a. Describe immunogenic responses by dose, over time from ELISA data.
b. Individual responses will be described over time and stratified by dose cohort.
c. Analysis will be performed using either mixed models or GEE.

In addition, models that account for correlation of responses within participant (such as marginal models or mixed models) will be used to study the immune responses. The approaches to missing data and the particular methods for modeling will be described in the analysis. Listings will show the observed data and, if applicable, imputed values and the approaches taken for imputation.

Should the need arise for terminating the study early, the investigative team will discuss with the SMC the reason for termination and determine which study questions can be addressed in an unbiased manner with the available data. The available data will be analyzed and interpreted in light of early termination.

9.1.1 Safety

The primary safety endpoint is the frequency of vaccine-related AEs, as classified by both intensity and severity through active and passive surveillance. Separate assessments of systemic and local reactions will be performed.

9.1.2 Immunogenicity Analysis

The primary immunogenic endpoint is antibody concentration as measured by ELISA at Day 70. Anti-$MSP1_{42}$ antibody will be measured by ELISA on Days 0, 14, 28, 42, 56, 70, 84, 140 and 238 as listed in the schedule of visits.

9.2 Sample Size

A group size of 15 participants per dose gives a power of about 0.8 for detecting one or more serious or severe AE that occur with a probability of 0.1 per participant.

Based on an analysis of the human antibody responses to a number of malaria antigens that have been tested in clinical trials (the three components of Combination B [13] and RTS,S [14]), the observed coefficient of variation in the range of antibody concentrations has been found to be relatively constant at approximately 1.2 to 1.4. However, using the day 42 human antibody data from the ongoing U.S. Phase 1 vaccine trial of the individual $MSP1_{42}$ vaccines (unpublished data), the coefficient of variation was found to be slightly higher than what has been observed in the other trials. A mean of 1.7 was found for the coefficient of variation from the $MSP1_{42}$-3D7 vaccine and a mean of 1.8 for the $MSP1_{42}$-FVO vaccine. Given the coefficient of variation of 1.7, and assuming that one group has mean antibody concentrations that are at least 2.4 times the mean antibody concentrations of the other group, then the two-sided Mann-Whitney test assuming a significance of 0.05 performed on the sample size of 30 per group (See Secondary Objective 1, Section 9.1) will have a power of greater than 0.8. A slightly higher coefficient of variation of 1.8 would detect a difference of 2.5 times, using the same sample size, significance and power assumptions. These estimates are similar to those computed for a stratified t-test (ANOVA) with the sample size adjusted assuming an asymptotic relative efficiency of 0.955.

10.0 PROTECTION OF HUMAN SUBJECTS

10.1 Institutional Review Board/Ethics Committee

The Investigator will be responsible for obtaining IRB approval for the study. Before the start of the study, the appropriate documents (including the Protocol, Investigator's Brochure, Informed Consent Form, information sheets, and advertisements) will be submitted to the IRBs. A copy of the study approval (including approval of the informed consent form) is to be maintained in the Investigator's study document binder and a copy will be supplied to the Sponsor. During the study, the Investigator is responsible for providing the IRBs with all documents subject to review (i.e., protocol amendments, informed consent form updates, advertisements, and any written information that may be provided to the subject). Annual reports on the progress of the study will be made to the IRBs by the Investigator in accordance with IRB guidelines and government regulations.

Informed Consent:

In obtaining and documenting informed consent, the Investigator must comply with the applicable regulatory requirements, Good Clinical Practice (GCP) and ethical principles. The written informed consent form must be approved by all participating Institutional Review Boards (IRB) prior to its use.

Risks:

Risks to the participants are associated with venipuncture and with immunization. These risks are outlined below.

Female participants will be cautioned of the unknown risk of study vaccines to the fetus and will be advised to use adequate birth control methods for the duration of the study.

Venipuncture

Risks occasionally associated with venipuncture include pain, bruising, and infection at the site of venipuncture, lightheadedness, and syncope (rarely).

Immunization

Possible local vaccine reactions include pain, swelling, erythema, induration, limitation of limb movement for several days, lymphadenopathy or pruritus at the injection site. Local SC nodules, believed to be granulomatous reactions to aluminum hydroxide, have been observed with use of aluminum hydroxide-based adjuvants. Thus, most aluminum hydroxide-adsorbed vaccines are injected IM rather than SC. Systemic reactions such as fever, chills, headache, fatigue, malaise, myalgia and joint pain may also possibly occur. Immediate hypersensitivity reactions including urticaria, anaphylaxis or other IgE mediated responses are possible as with any vaccine. As with any investigational vaccine, there is a theoretical possibility of risks about which there is no present knowledge. Participants will be informed of any such risks should further data become available.

10.4 Benefits

Participants will not receive any direct benefit from participation in this study. It is hoped that information gained in this study will contribute to the development of a safe and effective malaria vaccine.

10.5 Confidentiality:

All study-related information will be stored securely at the study site. All participant information will be stored in locked file cabinets in areas with access limited to study staff. All laboratory specimens, reports, study data collection, process and administrative forms will be identified by coded number only to maintain participant confidentiality. All computer entry will be done by coded number only, and all local databases will be secured with password-protected access systems. Forms, lists, logbooks, appointment books and any other listings that link participant ID numbers to other identifying information will be stored in a separate, locked file in an area with limited access.

Participants' study information will not be released without the written permission of the participant, except as necessary for monitoring by NIAID or Coley Pharmaceutical Group and/or their contractors and the FDA.

10.6 Compensation:

Participants will be paid $50.00 for their screening visit and $75.00 per visit during participation in the study (18 visits at $75.00 = $1,350.00, plus $50.00 for screening = $1400.00 total) for

their time, travel, and inconvenience. Participants will be paid for the screening visit and for each clinic visit if enrolled. Volunteers who attend as alternates on the day of first vaccination, but are not enrolled, will receive payments for the screening visit and for one clinic visit. A bonus of $200.00 will be paid for completion of all visits. The total payment will be divided over the course of the study with the bonus dispensed upon completion of the trial.

11.0 REFERENCES

1. World Health Organization, Tropical Disease Research. Source: World Health Report 2002. http://www.who.int/tdr/diseases/malaria/diseaseinfo.htm.

2. Good, M.F., Kaslow, D.C. & Miller, L.H. Pathways and strategies for developing a malaria blood-stage vaccine. Annu. Rev. Immunol. 16:57-87 (1998).

3. Holder A.A. & Freeman R.R. Protective antigens of rodent and human bloodstage malaria. Philos Trans R Soc Lond B Biol Sci. 307:171-7 (1984).

4. McBride J.S. & Heidrich H.G. Fragments of the polymorphic Mr 185,000 glycoprotein from the surface of isolated Plasmodium falciparum merozoites form an antigenic complex. Mol Biochem Parasitol. 23:71-84 (1987).

5. Gerold P, Schofield L, Blackman MJ, et al. Structural analysis of the glycosyl-phosphatidylinositol membrane anchor of the merozoite surface proteins-1 and -2 of Plasmodium falciparum. Mol Biochem Parasitol. 75:131-43 (1996).

6. Haldar K, Ferguson MA, Cross GA. Acylation of a Plasmodium falciparum merozoite surface antigen via sn-1,2-diacyl glycerol. J Biol Chem. 260:4969-74 (1985).

7. Holder AA, Blackman MJ, Burghaus PA, et al. A malaria merozoite surface protein (MSP1)-structure, processing and function. Mem Inst Oswaldo Cruz. 87 Suppl 3:37-42 (1992).

8. Blackman MJ, Heidrich HG, Donachie S, et al. A single fragment of a malaria merozoite surface protein remains on the parasite during red cell invasion and is the target of invasion-inhibiting antibodies. J Exp Med. 172:379-82 (1990).

9. Cody,C.L., Baraff,L.J., Cherry,J.D., Marcy,S.M. & Manclark,C.R. Nature and rates of adverse reactions associated with DTP and DT immunizations in infants and children. *Pediatrics* 68, 650-660 (1981).

10. Merck and Co.,I. Recombivax HB Product Insert. http://www.whale.to/m/recombivax_hb.html . 1999. 2-14-2002.
Ref Type: Electronic Citation

11. Cooper CL, Davis HL, Morris ML, Efler SM, Adhami MA, Krieg AM, Cameron DW, Heathcote J. CPG 7909, an immunostimulatory TLR9 agonist oligodeoxynucleotide, as adjuvant to Engerix-B HBV vaccine in healthy adults: a double-blind phase I/II study. *J Clin Immunol.* 2004 Nov;24(6):693-701.

12. Cooper CL, Davis HL, Morris ML, Efler SM, Krieg AM, Li Y, Laframboise C, Al Adhami MJ, Khaliq Y, Seguin I, Cameron DW. Safety and immunogenicity of CPG 7909 injection as an adjuvant to Fluarix influenza vaccine. *Vaccine.* 2004 Aug 13;22(23-24):3136-43.

13. Saul, A., Lawrence G., Smillie A., et al. Human Phase 1 vaccine trials of 3 recombinant asexual stage malaria antigens with Montanide ISA 720 adjuvant. *Vaccine* 17:3145-3159 (1999).

14. Stoute,J.A. *et al.* A preliminary evaluation of a recombinant circumsporozoite protein vaccine against Plasmodium falciparum malaria. RTS,S Malaria Vaccine Evaluation Group. *N. Engl. J. Med.* 336, 86-91 (1997).

APPENDIX A: SCHEDULE OF VISITS

		Month		0					1					2					3	5	8
Procedures	Blood Volume[1]	Day	Pre	0	1	3	7	14	28	29	31	35	42	56	57	59	63	70	84	140	236
Obtain Informed Consent			X																		
Complete History & Physical			X																		
Interim Clinical Evaluation				X	X	X	X	X	X	X	X	X	X	X	X	X	X	X	X	X	X
CBC & differential[2]	4 mL		X	X		X	X	X	X		X	X	X	X		X	X	X	X	X	X
ALT/creatinine	5 mL		X	X		X		X	X		X		X	X		X		X			
Urinalysis			X	X				X	X				X	X				X	X		X
Urine pregnancy test (females only)			X	X					X					X							
HIV, HCV, HBsAg	5 mL		X																		
Anti-dsDNA, (Rheumatoid factor, ANA, anti-ssDNA[3])	7.5 mL		X	X			X		X			X		X			X		X		X
C3, C4, CH50	5 mL			X																	
VACCINATION				X					X					X							
Anti-MSP1 antibody ELISA	10 mL			X				X	X				X	X				X	X	X	X
Growth Inhibition Assay	20 mL			X									X	X				X			X
T/B cell analysis	20 – 40mL			40mL			40mL		20mL		20mL	20mL		20mL		20mL	20mL			20mL	20mL
Total Blood Volume (mL)			21.5 mL	91.5mL		9mL	51.5 mL	19mL	46.5mL		29mL	36.5mL	39mL	66.5mL		29mL	31.5mL	39mL[4]	21.5mL	34mL	61.5mL

1. Total blood volume drawn over the course of the study is 626.5 mL.
2. CBC parameters to be assessed for safety: WBC, ANC, hemoglobin, platelet count
3. ( ) indicates that serum aliquots will be withheld for these tests should the anti-dsDNA be positive. ANA and Rheumatoid factor will be done at screening and on day 0.
4. On selected consenting volunteers, plasmapheresis will be performed

APPENDIX B - TOXICITY TABLE FOR GRADING LABORATORY ADVERSE EVENTS

These tables are to be used to assess laboratory adverse events for those tests to be performed as part of the $MSP1_{42}$-C1 /Alhydrogel® +/- CPG 7909 malaria vaccine clinical trial protocol.

ABBREVIATIONS: Abbreviations utilized in the Table:
ULN = Upper Limit of Normal
LLN = Lower Limit of Normal

ESTIMATING SEVERITY GRADE

GRADE 1 **Mild:** no effect on activities of daily living; no medical intervention/therapy required
GRADE 2 **Moderate:** partial limitation in activities of daily living (can complete ≥ 50% of baseline); no or minimal medical intervention/therapy required
GRADE 3 **Severe:** activities of daily living limited to < 50% of baseline; medical evaluation/therapy required

**Laboratory**	**Mild (Grade 1)**	**Moderate (Grade 2)**	**Severe (Grade 3)**
Hgb (♀) – decrease from testing laboratory LLN in gm/dl	≥ 1.0 & < 1.5	≥ 1.5 & < 2.0	≥ 2.0
Hgb (♂) – decrease from testing laboratory LLN in gm/dl	≥ 1.5 & < 2.0	≥ 2.0 & < 2.5	≥ 2.5
WBC – cells/mm^3 (Increase in WBC)	≥ 11000 & < 15000	≥ 15000 & < 20000	≥ 20000
WBC – cells/mm^3 (Decrease in WBC)	< 3500 & ≥ 2500	< 2500 & ≥ 1500	< 1500
ANC – cells/mm^3 (Decrease in ANC)	< 1500 & ≥ 1000	< 1000 & ≥ 500	< 500
Platelets – cell/mm^3	< 135,000 & ≥ 125,000	< 125,000 & ≥ 100,000	< 100,000
ALT (increase by factor)	> 1.0 & < 2.5 x ULN	≥ 2.5 & < 4 x ULN	≥ 4 x ULN
Serum creatinine – mg/dL	1.1 – 1.5 x ULN	1.6 – 2.9 x ULN	≥ 3.0 x ULN

**URINALYSIS**			
	Grade 1	Grade 2	Grade 3
Proteinuria	2+<br>or<br>0.5 - 1 gm loss/day	3+<br>or<br>1 - 2 gm loss/day	4+<br>or<br>2 - 3.5 gm loss/day
Hematuria	5-10 rbc/hpf	>10 rbc/hpf	gross, with or without clots, OR red blood cell casts

APPENDIX C – TOXICITY TABLE FOR GRADING UNEXPECTED SYSTEMIC ADVERSE EVENTS

These tables are to be used to grade unexpected adverse events not described in Table 6-2 of this protocol.

**Vital Signs***	**Mild (Grade 1)**	**Moderate (Grade 2)**	**Severe (Grade 3)**
Tachycardia – beats per minute	110-120	120-140	>140
Bradycardia – beats per minute	50-54	45-49	<45
Hypertension (systolic) – mm Hg (with repeat testing at same visit)	141-150	151-155	>155
Hypertension (diastolic) – mm Hg (with repeat testing at same visit)	91-100	100-110	>110
Hypotension (systolic) – mm Hg (with repeat testing at same visit)	85-89 (and symptomatic)	80-84 (and symptomatic)	<80
* Participant should be at rest for measurement of vital signs			

**Systemic**	**Mild (Grade 1)**	**Moderate (Grade 2)**	**Severe (Grade 3)**
Anorexia	Loss of appetite without decreased oral intake lasting greater than 48 hours	Loss of appetite associated with decreased oral intake without significant weight loss	Loss of appetite associated with significant weight loss
Vomiting	1-2 episodes/24 hours	> 2 episodes/24 hours	Prevents daily activity, requires outpatient IV hydration
Diarrhea	2-3 loose stools/24 hours	4-5 loose stools/24 hours	>6 loose stools or requires outpatient IV hydration
Constipation	NA	Persistent constipation requiring regular use of dietary modifications, laxatives, or enemas	Obstipation with manual evacuation indicated
Fatigue	No interference w/activity	Some interference w/activity	Significant, prevents daily activity
Arthritis	Mild pain with inflammation, erythema or joint swelling – but not interfering with function	Moderate pain with inflammation, erythema or joint swelling – interfering with function, but not with activities of daily living	Severe pain with inflammation, erythema or joint swelling –and interfering with activities of daily living

Mucocutaneous Reaction/Rash	Erythema; pruritus or localized macular rash	Diffuse, maculo-papular rash, dry desquamation	Vesiculation or moist desquamation or ulceration
Vasovagal episode (associated with a procedure of any kind)	Present without loss of consciousness	Present with transient loss of consciousness	NA
Vertigo	Causes no or minimal interference with usual daily activities	Causes greater than minimal interference with usual daily activities	Inability to perform daily activities
Cough	transient- no treatment	persistent cough; treatment responsive	Paroxysmal cough; uncontrolled with treatment
Bronchospasm, Acute	transient; no treatment; 70% - 80% FEV_1 of peak flow	requires treatment; normalizes with bronchodilator; FEV_1 50% - 70% (of peak flow)	no normalization with bronchodilator; FEV_1 25% - 50% of peak flow; or retractions present
Dyspnea	dyspnea on exertion	dyspnea with normal activity	dyspnea at rest
Hypersensitivity	Transient flushing or rash	Rash; flushing; urticaria; dyspnea	Symptomatic bronchospasm, with or without urticaria; parenteral medications(s) indicated; allergy-related edema/angioedema; hypotension
Illness or clinical adverse event NOT identified on the toxicity table	No interference w/activity	Some interference w/activity	Prevents daily activity and requires medical intervention

APPENDIX D – MALARIA INFORMED CONSENT COMPREHENSION TEST

Johns Hopkins University / Bloomberg School of Public Health
Center for Immunization Research

Malaria MSP 1 Comprehension Exam

Date: _______________ Vol Initials: ________ Screen # _____________

1. **As part of this study, you will be injected with a live malaria parasite………T F**
2. **This vaccine will protect you from getting malaria……………………………...T F**
3. **There is a chance you could have local reactions (pain, redness, swelling, itching) at the site of the injection………………………………………………T F**
4. **Women enrolled in this study must not be pregnant or nursing………………..T F**
5. **If you change your mind about being in the study after you are vaccinated, you can withdraw your consent…………………………………………………T F**
6. **This vaccine has been given to hundreds of people already, so we know it is completely safe………………………………………………………………T F**
7. **You will receive an injection of the same vaccine once monthly for three months…………………………………………………………………………..T F**
8. **If you feel sick during the study, you should keep it to yourself…………………T F**
9. **If you join this study, you will need to be followed for a total of 8 months…….. T F**
10. **Before joining the study you will be tested for HIV, Hepatitis B and Hepatitis C……………………………………………………………………..T F**
11. **You will fill out a diary card for 6 days after each vaccination…………………T F**

12. **It is OK to enroll in other investigational agent studies while you are still in this study……………………………………………………………………….T F**

13. **It is important for you to stay in the clinic for 60 minutes after each injection…T F**

14. **You will have blood drawn during most of your clinic visits……………………..T F**

15. **You can not get malaria from this vaccine study………………………………….T F**

Total # correct before review:_______ Total # correct after the review: __________

Reviewed by: ________________________________ Date: ________________

4/20/05 gk
